# Depolymerization of Commercial Polymethacrylates Triggered by Hydroperoxides

**DOI:** 10.1021/jacs.6c10099

**Published:** 2026-08-03

**Authors:** Ferdinando De Luca Bossa, Matylda Szewczyk-Łagodzińska, Alessandro Magro, Halil Ibrahim Coskun, Gorkem Yilmaz, Ting-Chih Lin, Elizabeth DiFiglia, Krzysztof Matyjaszewski

**Affiliations:** Department of Chemistry, 6612 Carnegie Mellon University, 4400 Fifth Avenue, Pittsburgh, Pennsylvania 15213, United States

## Abstract

The chemical recycling of poly­(methyl methacrylate) (PMMA) and related polymethacrylates is challenging due to the all-carbon backbone, which resists thermal and chemical degradation under mild conditions. Existing depolymerization strategies either require modification of the PMMA structure or rely on photochemical activation, limiting versatility and practical scalability. Herein, we report a thermally driven, hydrogen-atom-transfer (HAT)-initiated depolymerization of unmodified commercial polymethacrylates. Simple hydroperoxides, *tert*-butyl hydroperoxide (TBHP) and cumene hydroperoxide (CHP), serve as radical precursors, requiring no photoactivation, no specialized chain ends, and no chlorinated solvents. At elevated temperatures, oxygen-centered radicals are generated and abstract hydrogen atoms from the polymer chains. This triggers β-fragmentation and initiates chain unzipping. At 220 °C with 10 mol % TBHP, 30% of the polymer remained, indicating 70% depolymerization. However, only 55% was in the form of intact monomer, and 15% was consumed by side reactions during long exposure to high temperature. This approach was extended to poly­(butyl methacrylate), poly­(benzyl methacrylate), and poly­(phenyl methacrylate), and demonstrated moderate tolerance to ambient oxygen levels. These results establish thermally triggered HAT depolymerization as a simple approach for the depolymerization of commercial polymethacrylates.

## Graphical abstract

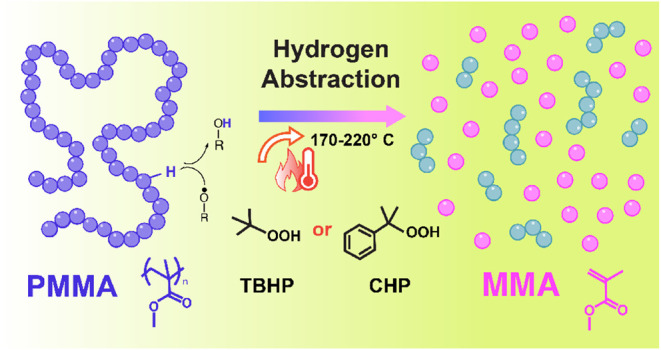

## Introduction

Poly­(methyl methacrylate) (PMMA), commercially known as Plexiglas or Perspex, is among the most versatile engineering thermoplastics. Its optical clarity, lightweight, and biocompatibility enable applications in the optics, construction, and biomedical sectors. Despite global PMMA production reaching approximately 2.6 million metric tons in 2025,^1^ its recycling remains challenging, owing to its robust all-carbon backbone.^2–5^ The most established chemical recycling route for PMMA is pyrolysis: high-temperature thermal decomposition in the absence of oxygen.^6–16^ Pyrolysis of PMMA proceeds via chain-end and backbone scission, generating radicals that drive recovery of monomer in >95% yield (Scheme 1A). While defect-containing chains can depolymerize at lower temperatures, high monomer recoveries require random backbone scission, which occurs only at elevated temperatures. As a result, pyrolysis typically requires temperatures exceeding 400 °C and specialized equipment. Simple benchtop microdistillation setups can achieve only ∼50% monomer recovery. The energy demand and infrastructure requirements of pyrolysis-based recycling limit its viability for decentralized or small-scale applications.

**Scheme 1:**
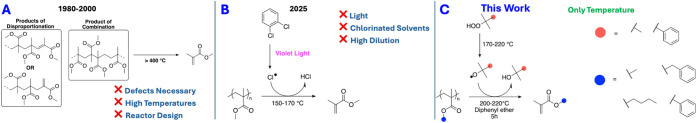
Comparison of Commercial PMMA Depolymerization/Degradation Strategies: (A) Defect-Triggered Depolymerization;^16^ (B) Photo HAT-Triggered Depolymerization;^58^ (C) Thermal HAT-Triggered Depolymerization. Red Dot: Me for TBHP and Ph for CHP

Reversible deactivation radical polymerization techniques^17^ have previously been exploited to lower the temperature required for radical formation and depolymerization initiation. Two main approaches were employed: atom transfer radical polymerization (ATRP)^18^ and reversible addition–fragmentation chain transfer (RAFT)^19^ polymerization. These techniques allow for the introduction of tailored chain ends or pendant functionalities. Once activated, these groups initiate depolymerization under milder conditions. The generated radical is involved in a reversible equilibrium between polymerization and depolymerization. This equilibrium is governed by thermodynamic parameters, namely, the equilibrium monomer concentration ([*M*]_eq_) and the ceiling temperature (*T*_c_) (eqs S2 and S3). [*M*]_eq_ and *T*_c_ define the monomer concentration and temperature at which the two processes are balanced. By operating below [*M*]_eq_ or above *T*_c_, the equilibrium is shifted toward depolymerization.^20–28^ Depolymerization strategies have been successful in solution^29–37^ and in bulk,^38–45^ in the presence of oxygen,^46–48^ under continuous flow,^49^ or with external stimuli. Such stimuli include light irradiation,^50–54^ microwave,^55^ or electrochemical^56,57^ assistance.

A fundamental limitation of RDRP approaches is the requirement for “custom-made” functionalities, which are absent in commercial PMMA. This restricts the applicability of direct recycling of the vast majority of commercial PMMA. Recent photochemical hydrogen-atom transfer (HAT) strategies were developed to address this gap.^58,59^ UV or visible-light irradiation of solutions of PMMA in chlorinated solvents and/or substituted aromatic solvents was used to generate radicals in situ. These radicals abstract hydrogen atoms from commercial PMMA, triggering chain fragmentation and depolymerization (Scheme 1B).

These methods rely on short-wavelength UV/violet light and chlorinated solvents. Short-wavelength light has poor penetration, and chlorinated solvents do not align with the green depolymerization goals. Although non-chlorinated media were previously tested, they resulted in less efficient depolymerization.^59^ No existing strategy for the depolymerization of commercial PMMA combines thermal activation with operational simplicity. We therefore decided to develop a HAT-based depolymerization of commercial polymethacrylates driven solely by temperature (Scheme 1C).

The key thermodynamic parameter in HAT is the bond dissociation energy (BDE) difference between the cleaved and formed bonds.^60–66^ The other requirement is the polarity of the radical, which needs to be electrophilic, or in other words, hydrogenophilic, for the process to be kinetically favored.^67^ C–H BDEs typically range from 80 to 100 kcal/mol,^68^ with benzylic and allylic positions at the lower end and saturated alkanes at the upper end. PMMA has three abstraction sites: backbone CH_2_, α-methyl CH_3_, and ester CH_3_, all calculated to lie near the upper BDE range (∼100 kcal/mol).^69^ Oxygen-centered radicals are particularly attractive abstractors because the O–H bond is especially strong (BDE ∼105 kcal/mol), providing a thermodynamic driving force for hydrogen abstraction.^69^ Hydroperoxides (HP) are inexpensive, industrially available, and thermally labile, making them promising candidates for radical generation at elevated temperatures. We selected *tert*-butyl hydroperoxide (TBHP) and cumene hydroperoxide (CHP) on the basis of their high 10-h half-lifetime temperatures (170 °C for TBHP^70^ and 155 °C for CHP^71,72^ ). At higher temperatures, the half-lives of these hydroperoxides are significantly shorter. For example, at 200 and 220 °C, the half-lives of TBHP are ∼30 and 6 min.^70^

Herein, we report the thermally driven, HAT-initiated depolymerization of unmodified, commercially available PMMA and related polymethacrylates using TBHP and CHP as radical precursors (Scheme 1C). This strategy requires no photochemical activation, no customized functionalities, no chlorinated solvents, and operates under conditions that are moderately oxygen-tolerant. We systematically investigated the effects of temperature, hydroperoxide loading, and polymer concentration on depolymerization. We correlated chain fragmentation events with experimental depolymerization efficiency. The scope was extended to poly­(butyl methacrylate) (PBMA), poly­(benzyl methacrylate) (PBzMA), and poly­(phenyl methacrylate) (PPhMA), revealing how pendant ester group structure affects abstraction and β-fragmentation selectivity. Finally, we identified the key mechanistic bottlenecks: alkoxyl radical decomposition, competing radical termination, and undesired monomer consumption. We therefore outlined strategies for future optimization toward efficient thermal depolymerization of commercial polymethacrylates.

## Results and Discussion

### TBHP-Mediated Depolymerization of PMMA

Depolymerization experiments were conducted at [MMA]_eq_ or below and at different temperatures. [MMA]_eq_ was calculated as 0.340, 0.912, and 1.7 M at 170, 200, and 220 °C, respectively, based on Δ*H* ^0^ = −59.4 kJ mol^–1^ and Δ*S* ^0^ = −124.1 J mol^–1^ K^–1^ (eqs S2 and S3 in the Supporting Information).^73^ Since [*M*]_eq_ increases with temperature (eqs S2 and S3), raising the temperature allows for depolymerization in more concentrated polymer solutions. The PMMA used (purchased from Beantown Chemical) had initial *M*_n_ = 180 kDa, *M*_p_ = 400 kDa, and *Đ* = 2.6.

The reaction efficiency was evaluated by monitoring the shift in the peak molecular weight (*M*_p_) via GPC and the monomer yield by ^1^H NMR. We selected *M*_p_ rather than *M*_n_ because *M*_p_ reflects the most abundant chain population and could therefore provide more representative and robust metrics for calculating the number of fragmentation events. The longer chains contain more abstractable C–H bonds and therefore statistically should be more likely to undergo H-abstraction and fragmentation. At a fixed [TBHP]/[MMA]_RU_ molar ratio of 5 mol %, the reaction at 170 °C resulted in limited chain scission, with no significant monomer formation over 5 h. Additional experiments at [TBHP]/[MMA]_RU_ 5–10 mol % vs [MMA]_RU_ at reduced [MMA]_RU_ did not show any monomer formation (Figures S2–S7). In comparison, reactions at 200 and 220 °C resulted in a substantial decrease in *M*_p_ and increased monomer yields that plateaued at ∼45% and ∼50%, respectively, after the same time (Figures S2 and S3). In the absence of TBHP, no depolymerization or monomer formation was observed at 220 °C (Figure S8).

Motivated by the ∼50% monomer formation, we evaluated a range of [TBHP]: 0.1−10 mol % TBHP relative to [MMA]­RU at 200 °C and 1−50 mol % at 220 °C. Varying [TBHP] did not enhance monomer yield, which remained ∼40% at 200 °C and ∼50% at 220 °C (Figures S9 and S10). At higher hydroperoxide loadings and elevated temperatures, additional resonances appeared in the ^1^H NMR spectra between 3.4 and 4.0 ppm (Figure S11). We identified these as products of reactions between radicals and monomers, either by addition or abstraction. Therefore, we will address those as “consumed monomer”, while unreacted monomer units are “intact monomer”. To quantify these species, we deconvoluted the broad peak in the 3.4–4.0 ppm region. Assuming a constant total concentration of MMA and PMMA units, we calculated the peak-area percentage ratios for the monomer (3.78 ppm) and the polymer (3.63 ppm) (Figure S12). Total depolymerization, including intact and consumed monomer, reached a maximum of 46% using [TBHP]/[MMA]_RU_ = 5 mol % at 200 °C (Figure 1B left). At 220 °C, total conversion was 71% using 10 mol % TBHP loading (Figure 1B, right). Across nearly all conditions, prolonged reaction times decreased the intact monomer concentration (Figure 1B).

**Figure 1:**
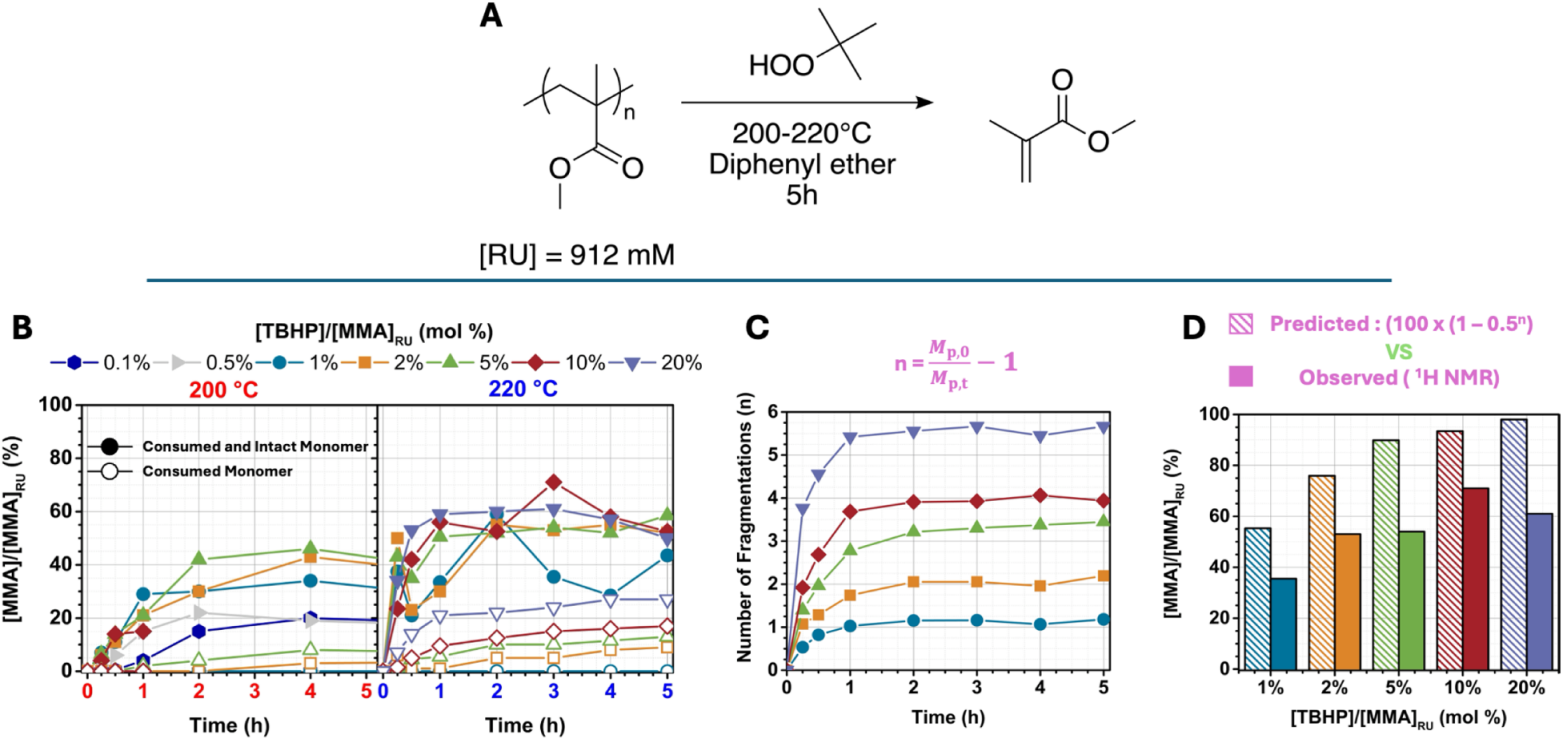
(A) Depolymerization reactions performed at 200 and 220 °C with different molar ratios [TBHP]/[MMA]_RU_, and fixed [MMA]_RU_ = 912 mM. (B) Kinetic analysis of depolymerization; total depolymerized PMMA (filled symbols) and consumed MMA (open symbols). (C) Average number of fragmentations of the polymeric chains (*n*), obtained with different mol % of TBHP at 220 °C, calculated from the ratio [(*M*_p,0_/*M*_p,t_) – 1] measured via GPC. (D) Comparison of predicted depolymerization based on the number of fragmentations (100­(1–0.5^n^)) and observed conversion, including intact and consumed monomer based on ^1^H NMR analysis, after 3 h at 220 °C.

After hydrogen abstraction and subsequent backbone fragmentation, only one of the two newly generated chain ends is active. Unzipping from a single active end can therefore deliver at most 50% depolymerization. Using the formula conversion = 100­(1 – 0.5^*n*^), where *n* is the average number of fragmentation events per chain, was obtained from the ratio $\left(\frac{M_{p,0}}{M_{p,t}}-1\right)$ (see Supporting Information), this value was converted into the theoretical depolymerization limit (Figure 1C and D, and eq S1.2). The gap between this theoretical value and the experimentally observed conversion is related to the contribution of radical termination. *t*-Butyloxyl radicals at elevated temperatures can decompose into acetone and methyl radicals (Scheme S1).^74,75^ The latter are nonproductive hydrogen abstractors, promoting termination and, therefore, competing with unzipping and suppressing overall depolymerization efficiency (vide infra and Supporting Information). Consistent with this picture, increasing TBHP loading widened the gap between theoretical and observed depolymerization, indicating more extensive termination.

### CHP-Mediated Depolymerization of PMMA

CHP was chosen as an alternative precursor for oxygen-centered radicals to maximize intact monomer formation and minimize monomer consumption. Upon homolysis, the cumyloxyl radical can fragment to either acetophenone and a methyl radical or to acetone and a phenyl radical (Scheme S2). Prior reports showed that phenyl radicals should not interfere with the depolymerization process (Scheme 1B).^58,59^ Thus, the latter pathway provides a potentially noninterfering radical that could reduce monomer consumption. Depolymerization with CHP at 220 °C was investigated over a range of [CHP]/[MMA]_RU_ loadings (1–50 mol %). As observed with TBHP, ^1^H NMR spectra showed emerging resonances in the 3.4–4.0 ppm region (Figure S13). We deconvoluted them to quantify intact and “consumed” monomer. At 20 mol % CHP, the total conversion reached 63%, comprising 41% intact monomer and 22% consumed monomer. The amount of the latter increased with increasing HP loading and time (Figure 2A). Overall, conversions plateaued at 50–60%, while the number of fragmentations per chain increased with CHP loading and exceeded that achieved with TBHP. This correlated with faster generation of cumyloxyl radicals from the less thermally stable CHP (Figure 2B). At the beginning of the reaction, no monomer is available; hence, the radicals can only terminate or react via HAT with the polymeric chains. The comparison of theoretical and observed depolymerization percentages revealed an increase in termination at higher CHP loadings, as in the TBHP case. This confirms that excessive radical concentration ultimately hinders unzipping (Figure 2C).

**Figure 2:**
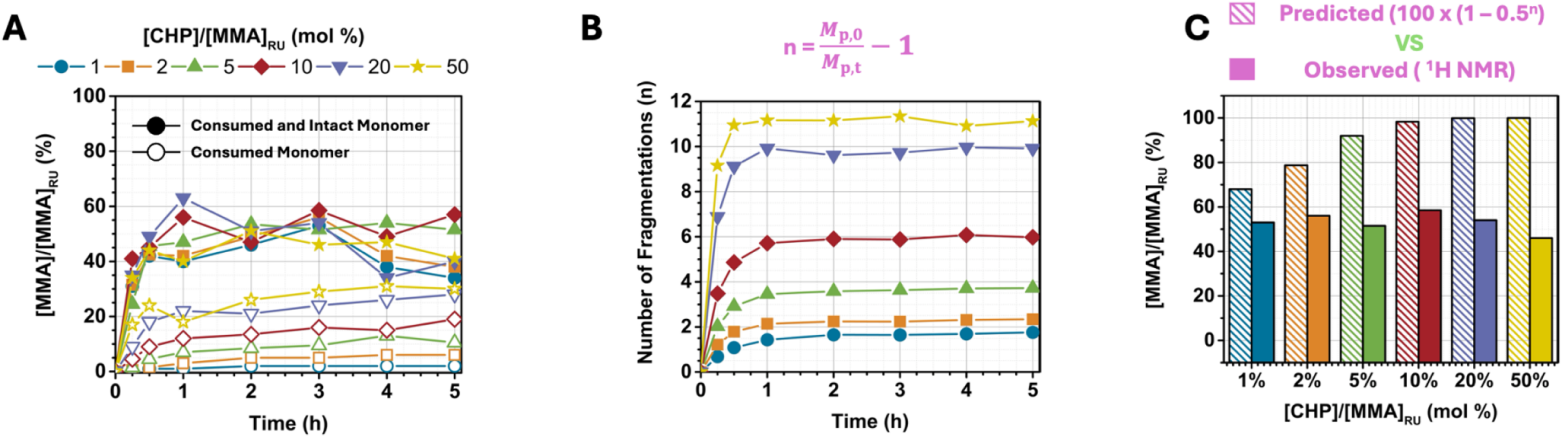
Kinetic analysis of depolymerization using CHP at 220 °C. (A) Total depolymerized PMMA (filled symbols) and consumed MMA (open symbols). (B) Average number of fragmentations of the polymeric chains (*n*), obtained with different mol % of CHP, calculated from the ratio [(*M*_p,0_/*M*_p,t_) – 1] measured via GPC. (C) Comparison of predicted (100­(1–0.5^n^)) and observed conversion, including intact and consumed monomer based on ^1^H NMR analysis.

Higher monomer and radical concentrations, caused by higher polymer concentration, also promote both termination and monomer consumption. Previous depolymerizations were operated at higher dilutions of 10 and 25 mM [MMA]_RU_ (i.e., 1–2.5 wt %).^58,59^ Therefore, we tested reactions at 2-fold (456 mM) and 18-fold (50 mM) dilutions of [MMA]_RU_ (Figure S14A and B). Dilution slowed both depolymerization and byproduct formation but did not improve overall performance. For example, after 3 h, total depolymerization reached only 35% at 50 mM, while at 912 mM it reached 59%. At both 456 mM and 50 mM, monomer formation increased systematically with initiator loading and reaction time, as it did at 912 mM (Figure S14). At the highest dilution (50 mM), however, the fraction of consumed monomer was markedly reduced—only ∼5% of generated monomer was consumed (Figure S14B). However, at such a low concentration, it was difficult to distinguish the consumed monomer species from the baseline noise (Figure S15).

### Oxygen Tolerance

The high concentration of methyl radicals could contribute to the oxygen tolerance. These radicals, formed before chain-end macroradicals and monomers, can capture the oxygen dissolved in the reaction mixture. Therefore, we performed reactions with TBHP and CHP (10 mol % relative to [MMA]_RU_) without deoxygenation and compared them with the deoxygenated ones. In both cases, the presence of oxygen did not significantly change monomer formation (Figure 3A and D). The depolymerization yields and number of fragmentations remained similar (Figure 3B, C ,E, and F). This shows moderate oxygen tolerance. The methyl radicals trapped O_2_, forming peroxyl radicals.

**Figure 3:**
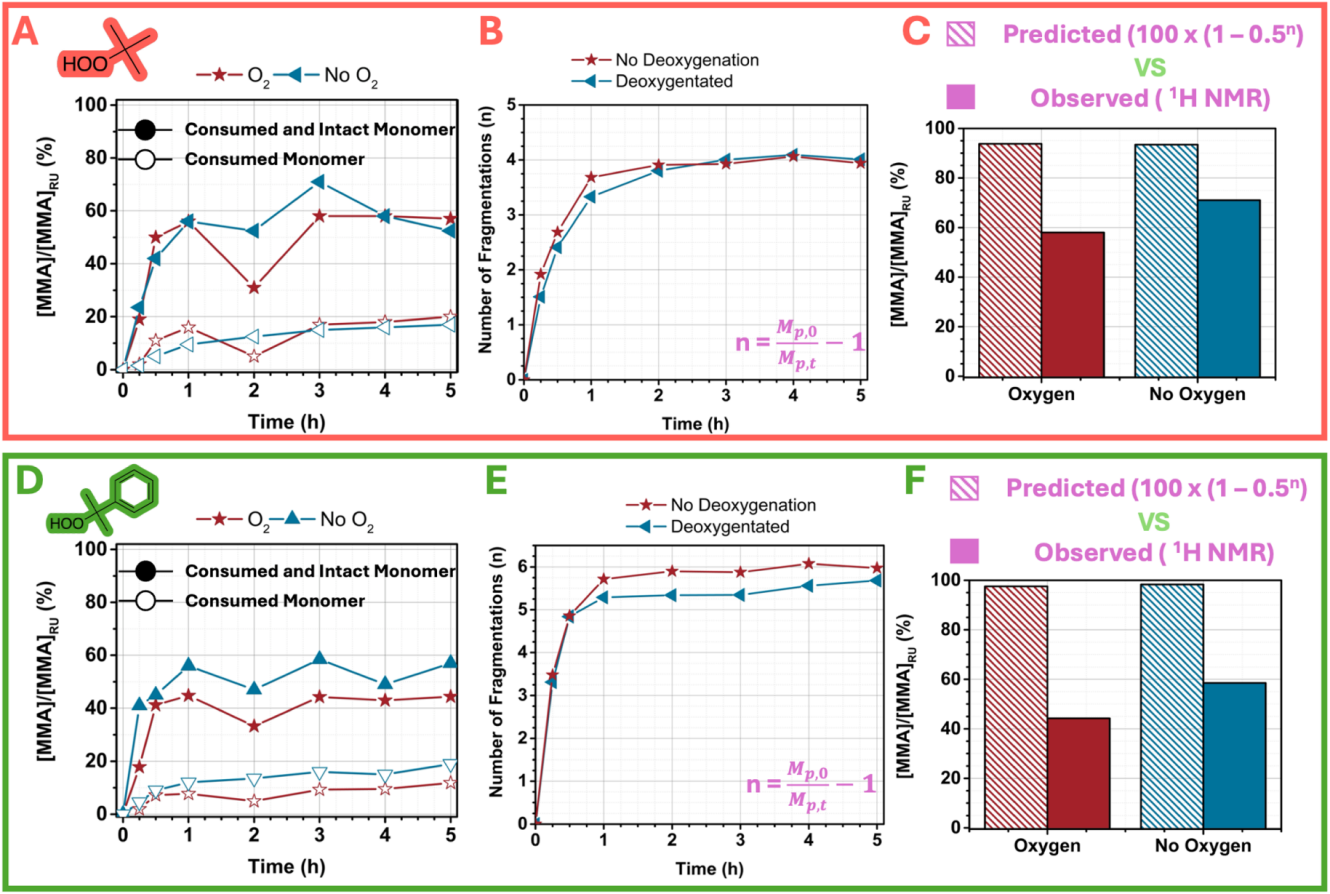
Depolymerizations performed at 220 °C, without degassing, with [HP]/[MMA]_RU_ = 10 mol % and fixed [MMA]_RU_ = 912 mM. (A, D) Kinetic analysis comparison of depolymerizations performed with and without degassing. (B, E) Average number of fragmentations of the polymeric chains (*n*), calculated from the ratio [(*M*_p,0_/*M*_p,t_) – 1] measured via GPC. (C, F) Comparison of predicted (100­(1–0.5^n^)) and observed depolymerization, including intact and consumed monomer based on ^1^H NMR analysis after 3 h.

### Polymer Scope

To broaden the polymer scope and gain insight into the abstraction/fragmentation mechanism, we applied our strategy to other polymethacrylates. PBMA, PBzMA, and PPhMA synthesized via free-radical polymerization (FRP) were selected as candidates for depolymerization (Figure S16).

Next, we performed depolymerizations under our optimized conditions (220 °C, 10 mol % HP relative to [*M*]_RU_). The experiments revealed that the extent of depolymerization was similar for all polymers. This confirms that high radical concentrations set a ceiling on depolymerization yield. For PBMA, we calculated the number of fragmentation events using both the apparent molar mass from conventional calibration using linear PMMA standards and the molar mass from universal calibration. We observed that *n* was essentially the same because it depends on the relative peak shift (Figure S17). Therefore, we used the apparent molar mass for the other polymers. The number of fragmentations followed the trend PMMA ≫ PBzMA > PBMA > PPhMA, with PMMA markedly outperforming other polymethacrylates (Figure 4C and F). To explain this behavior, we hypothesized that H-abstraction from the ester substituents can play an important role. As reported by Coote’s group,^69^ abstraction from the main chain −CH_2_ and substituent alkyl ester group is more favored than that from the main chain α-CH_3_. In this framework, PBMA offers two abstractable side-chain α-hydrogens instead of three for PMMA, which reduces fragmentation efficiency. The lower fragmentation efficiency may also be attributed to the presence of additional abstractable hydrogens in the *n*-butyl ester substituent. In PBzMA, abstraction should be more favorable because it yields a resonance-stabilized benzylic radical. However, this stabilization could reduce the fragmentation rate. In PPhMA, side-chain-driven fragmentation is not possible, consistent with the system having the lowest observed fragmentation and depolymerization.

**Figure 4:**
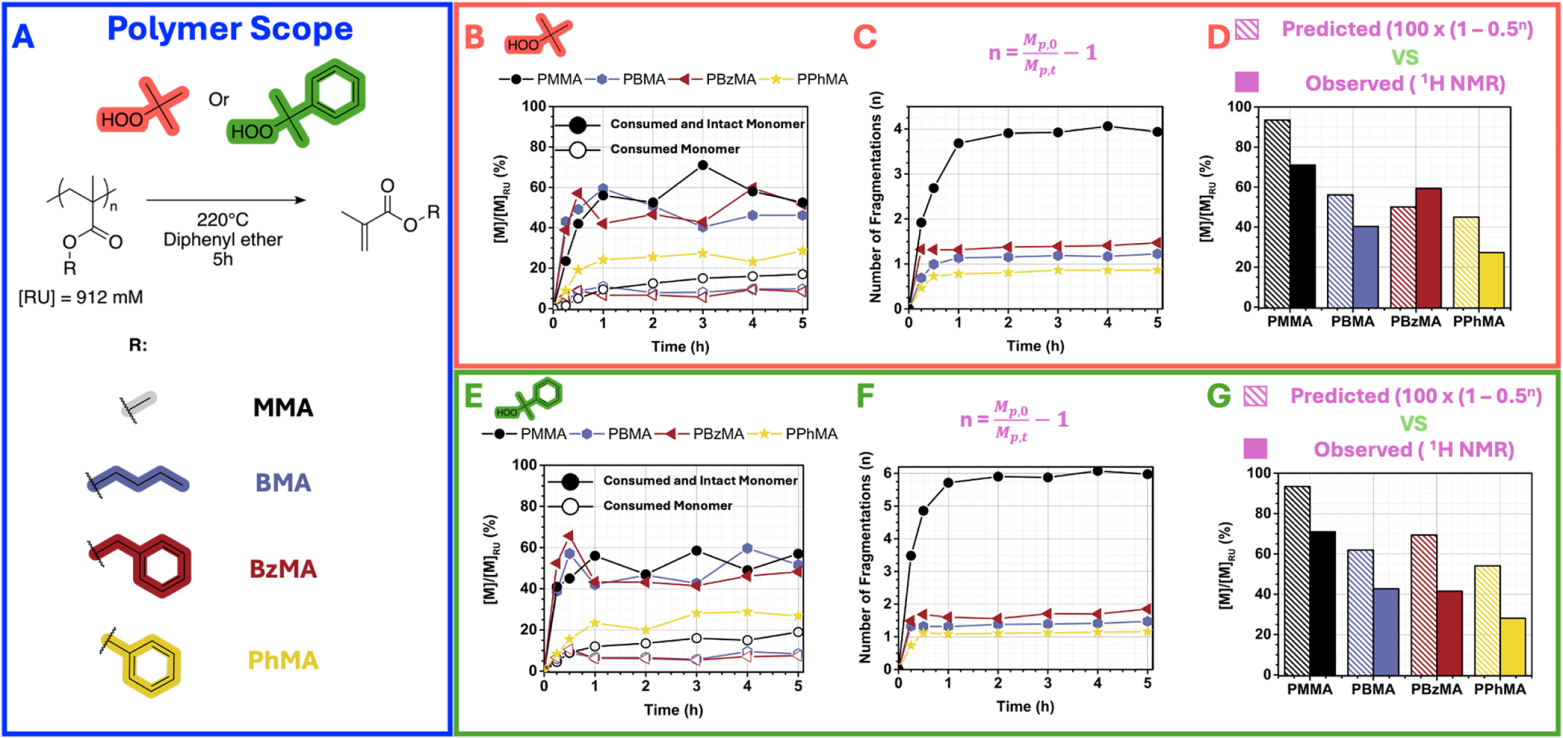
(A) Depolymerizations of polymethacrylates performed at 220 °C with [HP]/[MMA]_RU_ = 10 mol % and fixed [MMA]_RU_ = 912 mM. (B, E) Kinetic analysis comparison of depolymerizations of PMMA, PBMA, PBzMA, and PPhMA. (C, F) Average number of fragmentations of the polymeric chains (n), calculated from the ratio [(*M*_p,0_/*M*_p,t_) – 1] measured via GPC. (D, G) Comparison of predicted (100­(1–0.5^n^)) and observed depolymerization, including intact and consumed monomer based on ^1^H NMR analysis after 3 h.

We also tested a commercial Plexiglas sheet (see Supporting Information for details). The reaction under the optimized conditions, **[MMA]**_**RU**_ = 912 mM, 220 °C, **[TBHP]** = **10 mol %** versus **[MMA]**_**RU**_, showed monomer formation and consumption, similar to the originally studied commercial PMMA. The initial molar mass at the peak of Plexiglas was 1,800 kDa (vs 400 kDa for commercial), which resulted in a larger shift in the GPC traces upon depolymerization and indicated more efficient fragmentation of higher-molar-mass chains. However, the overall depolymerization extent was lower than that for lower-molar-mass PMMA. We could attribute this difference to stabilizers present in Plexiglas and to the much higher viscosity of the reaction mixture, both of which can affect the outcome (Figure S18).

### Insight into Monomer Consumption

At [MMA]_0_ < [MMA]_eq_, polymer formation is not thermodynamically possible. Therefore, we examined the direct consumption of MMA using both HPs (10 mol % vs MMA), with [MMA]_0_ = 912 mM at 220 °C. After 5 h, ∼50% of the MMA was consumed. ^1^H NMR analysis revealed behavior analogous to that observed in the depolymerization experiments, characterized by the appearance of multiple resonances in the 3.4–4.0 ppm region. GPC analysis confirmed the absence of any polymer with molar mass above 1 kDa (Figures S19 and S20; Tables S1 and S2). The observed monomer consumption could arise from the formation of very short oligomers and from hydrogen-abstraction products. This is followed by capping with methyl radicals derived from degradation of the alkoxy radical. GC–MS analysis of the reaction mixture with CHP (10 mol %, [MMA] = 912 mM) after 5 h at 220 °C confirmed the presence of acetophenone (Figures S21 and S22). This reveals that cumyloxyl radicals fragment to acetophenone and a methyl radical, similar to TBHP.^74^ Additional peaks in the GC–MS chromatogram were attributed to consumed monomer (Figure S21).

Methyl radical formation via β-scission of the alkoxyl radical is a key kinetic limitation of this method. Methyl radicals are nucleophilic and do not meet the polarity requirements for efficient hydrogen abstraction (see SI discussion).^67,74^ Instead of contributing to HAT, they react with macroradicals and monomer, thereby promoting termination and monomer consumption. Elevated reaction temperatures (200–220 °C) are required to accelerate HAT kinetics, rather than to overcome an intrinsic barrier to backbone fragmentation or unzipping. The competition between β-scission and HAT is temperature-dependent: β-scission becomes more favorable above 100 °C and reduces the fraction of alkoxyl radicals available for productive abstraction.

Our data indicate that alkoxyl radicals abstracted hydrogen atoms from all three accessible positions in the polymethacrylates, consistent with previously reported calculations.^69^ However, only a minority of the alkoxyl radicals led to hydrogen abstraction (Figure S23). This inefficiency can be caused by self-reaction of the hydroxyl radical with another HP molecule, generating a less reactive peroxyl radical. Moreover, consistent with prior work,^76,77^ fragmentation is favored when it converts a less stabilized radical into a more stabilized one (e.g., primary → secondary/tertiary). This rationalizes why abstraction from the side chain could be important: it first generates a primary radical, which could transform into a tertiary radical, plausibly via elimination of formaldehyde and CO. The latter, after subsequent fragmentation, should yield a tertiary chain-end radical capable of efficient depolymerization (Scheme S3). Further studies are needed to verify this hypothetical mechanism. High HP loadings (10–20 mol %) were required to generate sufficient radical concentrations for efficient hydrogen abstraction and fragmentation. However, this also increased the methyl radical concentration, increasing both radical–radical termination and undesired monomer consumption—a direct consequence of the β-scission/HAT interplay described above. Future work will therefore focus on milder, more controlled radical generation and orthogonal activation chemistries to selectively target chain scission while suppressing monomer loss.

## Conclusions

We report a thermally activated, oxygen-tolerant, HAT-driven depolymerization strategy that requires no photochemical stimuli. This method effectively converted commercial polymethacrylates to monomers using hydroperoxides. At 220 °C, TBHP and CHP generated oxygen-centered radicals that abstracted hydrogen atoms from polymethacrylates, triggering β-fragmentation and initiating chain unzipping. Approximately 70% of PMMA was depolymerized using 10 mol % [TBHP]/[MMA]_RU_. However, only ca. 50% of MMA remained intact, and 15% was consumed, forming oligomers. The number of fragmentation events per chain was experimentally measured and was smaller than the theoretical values. The difference is due to high instantaneous radical concentrations, which induce termination events and monomer consumption, preventing clean unzipping. CHP outperformed TBHP in promoting fragmentation, but similarly, at higher loadings, it enhanced termination. This emphasizes that efficient depolymerization requires tuning radical generation rates and selectivity to maintain sufficiently long-lived chain ends. Alternatively, the monomer can be removed from the mixture before it can react with excess radicals. This can enhance the abstraction efficiency and improve the recovery of pure, intact monomer.

Collectively, these findings establish thermally triggered HAT depolymerization as a potential chemical recycling method for polymethacrylates. They also underline key points for further optimization: control over radical generation, alternative abstractors with tailored selectivity, and efficient monomer removal.

## Supplementary Material



## Notes

General information, experimental procedures, ^1^H NMR spectra, GPC traces, GC-MS data, supplementary schemes, tables, and discussion (PDF)
