# Supplementary material for: Depolymerization of Commercial Polymethacrylates Triggered by Hydroperoxides
Source: J Am Chem Soc. 2026 Aug 3;148(31):33622–30. doi: 10.1021/jacs.6c10099 (PMC13474945; doi:10.1021/jacs.6c10099)
Supplement: Supplementary file 1 [file ja6c10099_si_001.pdf]

Supporting Information

Depolymerization of Commercial Polymethacrylates Triggered by Hydroperoxides

Ferdinando De Luca Bossa, Matylda Szewczyk-Łagodzińska, Alessandro Magro, Halil Ibrahim Coskun, Gorkem Yilmaz, Ting-Chih Lin, Elizabeth DiFiglia, Krzysztof Matyjaszewski*

Department of Chemistry, Carnegie Mellon University, 4400 Fifth Avenue, Pittsburgh, Pennsylvania 15213, United States

Table of Contents

Table of Figures 1

Materials and Methods 4

Instrumentation 4

Nuclear Magnetic Resonance (NMR) Spectroscopy. 4

Size-Exclusion Chromatography (SEC/GPC). 4

GC-MS Analysis. 4

Abstraction Efficiency Calculation 5

GPC analysis representative example 6

Equilibrium monomer concentration and ceiling temperature 6

Polymer Synthesis 7

Procedure for PBuMA, PPhMA and PBzMA synthesis 7

General depolymerization procedure 7

Quantification of depolymerization 8

Reaction of MMA and 10 mol % t-butyl hydroperoxide at 220°C below $[M]_{eq}$ 8

Reaction of MMA and 10 mol % cumene hydroperoxide at 220°C below $[M]_{eq}$ 8

Discussion on methyl radical capability of abstracting H-atoms from polymethacrylates 27

Table of Figures

Figure S1. Representative GPC analysis of the reaction at 220°C with different $[HP]/[MMA]_{RU}$ (mol %). 6

Figure S2. Decrease of peak molecular weight (M_p) with time at $[TBHP]/[MMA]_{RU}$ = 5 mol % (RU = Repeating Unit). The shaded area represents ± one standard deviation from experiments performed in triplicate (n = 3). 9
Figure S3. Kinetics of depolymerization at 170-220°C and $[TBHP]/[MMA]_{RU}$ = 5 mol %. 10
Figure S4. ^{1}H NMR spectra recorded during kinetic study of depolymerization at 170°C and $[TBHP]/[MMA]_{RU}$ = 10 mol %, $[MMA]_{RU}$ = 50 mM. 11
Figure S5. ^{1}H NMR spectra recorded during kinetic study of depolymerization at 170°C and $[TBHP]/[MMA]_{RU}$ = 5 mol %, $[MMA]_{RU}$ = 50 mM. 12
Figure S6. ^{1}H NMR spectra recorded during kinetic study of depolymerization at 170°C and $[TBHP]/[MMA]_{RU}$ = 10 mol %, $[MMA]_{RU}$ = 10 mM. 13
Figure S7. ^{1}H NMR spectra recorded during kinetic study of depolymerization at 170°C and $[TBHP]/[MMA]_{RU}$ = 5 mol %, $[MMA]_{RU}$ = 10 mM. 14
Figure S8. PMMA MW evolution ($[MMA]_{RU}$ = 912 mM) in diphenyl ether at 220°C after 5h. 15
Figure S9. Kinetics of depolymerization at 200°C and different molar ratios $[TBHP]/[MMA]_{RU}$ (0.1, 0.5, 1, 2, 5, 10, mol %). 16
Figure S10. Kinetics of depolymerization at 220°C and different molar ratios $[TBHP]/[MMA]_{RU}$ (1, 2, 5, 10, 20, 50 mol %). 17
Figure S11. Example of kinetic analysis of depolymerization at 220°C with $[TBHP]/[MMA]_{RU}$ = 20 mol % and $[MMA]_{RU}$ = 912 mM. The formation of additional peaks in the region 3.4-4.0 ppm is visible. ^{1}H NMR was taken in $CDCl_3$ at 0, 0.25, 0.5, 1, 2, 3, 4, 5 h. 18
Figure S12. Deconvolution example of the depolymerization reaction mixture after 5h at 220°C using $[TBHP]/[MMA]_{RU}$ = 20 mol %. 50% PMMA, 23 % intact MMA, 27 % consumed MMA. 19
Figure S13. Example of kinetic analysis of depolymerization at 220°C with $[CHP]/[MMA]_{RU}$ = 20 mol % and $[MMA]_{RU}$ = 912 mM. The formation of additional resonance in the region between 3.4-4.0 ppm is visible. ^{1}H NMR was taken in $CDCl_3$ at 0, 0.25, 0.5, 1, 2, 3, 4, 5 h 20
Figure S14. Depolymerization kinetics using CHP with different [RU] at 220°C. 21
Figure S15. ^{1}H NMR spectrum of depolymerization at 220°C with $[CHP]/[MMA]_{RU}$ = 10 mol % and $[MMA]_{RU}$ = 50 mM. 22
Figure S16. GPC of the polymethacrylates synthesized by FRP. 23
Figure S17. Comparison of the number of fragmentations calculated using linear PMMA standards and universal calibration. Mark-Houwink parameters used : $K_{PMMA} = 7.56 \times 10^{-5}$ dLg^{-1}, $\boldsymbol{\alpha}_{PMMA} = 0.731$, $K_{PBMA} = 9.64 \times 10^{-5}$ dLg^{-1}, $\boldsymbol{\alpha}_{PBMA} = 0.696$.[1] 24
Figure S18. Results of the depolymerization reaction using a Plexiglas sheet. 25
Figure S19. ^{1}H NMR spectra in $CDCl_3$ of MMA polymerization using 10 mol % [TBHP]/[MMA] at 220°, and [MMA] = 912 mM (time points at 0, 0.25, 0.5, 1, 2, 3, 4, 5 h). 29

Figure S20. ^{1}H NMR spectra in $CDCl_3$ of MMA polymerization using 10 mol % [CHP]/[MMA] at 220°, and [MMA] = 912 mM (time points at 0, 0.25, 0.5, 1, 2, 3, 4, 5 h). 30
Figure S21. GC-MS Chromatogram of the reaction mixture $[CHP]/[MMA]_{RU}$ = 10 mol %, $[MMA]_{RU}$ = 912 mM after 5h at 220°C. 31
Figure S22. Mass spectrum of the peak at 5 min corresponding to acetophenone (m.w. 120.4 g/mol).. 31
Figure S23. Abstraction efficiencies over time with different loadings of HP................ 32

Table S1. ^{1}H NMR results of MMA polymerization at 220°C, $[MMA]_{RU}$ = 912 mM, $[TBHP]/[MMA]_{RU}$ = 10%.. 29
Table S2. ^{1}H NMR results of MMA polymerization at 220°C, $[MMA]_{RU}$ = 912 mM, $[CHP]/[MMA]_{RU}$ = 10%. 30

Scheme S 1. Mechanism of decomposition of TBHP, and degradation of the corresponding alkoxyl radical.. 26
Scheme S 2. Mechanism of decomposition of CHP, and degradation of the corresponding alkoxyl radical.. 26
Scheme S 3. Proposed mechanism of the depolymerization of polymethacrylates using hydroperoxides as hydrogen abstractors... 33

Materials and Methods

Diphenyl ether was purchased from Fisher Chemical and used without further purification. Poly(methyl methacrylate) was purchased from Beantown Chemical and used without further purification. It was characterized via ^{1}H NMR and gel permeation chromatography (GPC) in THF, calibrated with PMMA standards. It had a molecular weight M_n = 180 kDa , M_p = 400 kDa and $Đ$ = 2.6. Coloured cast acrylic (poly(methyl methacrylate), PMMA) sheets (Lesnlok, ASIN: B0CZ5HX6VT; 5 × 7 in., 1/8 in. thick, 3 mm) were purchased from Amazon and used as received. The grey sheet was used for this study (M_n = 590,000 kDa, M_p = 1,800 kDa, $Đ$ = 2.2). *t*-Butyl hydroperoxide (TBHP) and cumene hydroperoxide (CHP) were purchased from Aldrich. The former was a solution 70 % w/w in water, while the latter was 80 % w/w in cumene. They were both used without further purification.

Instrumentation

Nuclear Magnetic Resonance (NMR) Spectroscopy.

^{1}H NMR spectra were recorded using a Bruker Advance 500 MHz NMR spectrometer. $CDCl_3$ was used as a solvent, and the residual $CHCl_3$ solvent signal served as a reference.

Size-Exclusion Chromatography (SEC/GPC).

The apparent number-average molar masses (M_n) and dispersity ($Đ$) of polymers and the samples from the kinetic analysis were measured relative to poly(methyl methacrylate) (PMMA) standards using an Agilent 1260 Infinity II isocratic pump, a column set containing 3 PSS analytical columns (GRAM Lux 10^2 , 10^3 , 10^4 , 10^5 Å pore sizes), and refractive index (RI) detector. The eluent was THF, operated at 35 °C with a flow rate of 1 mL min-1.

GC-MS Analysis.

GC-MS analysis was performed on an Agilent 6890-5973 GC-MS workstation. The GC column was a Hewlett-Packard fused silica capillary column crosslinked with 5% phenylmethylsiloxane. Helium was used as the carrier gas. The following conditions were used for all GC-MS analyses: injector temperature, 250 °C; initial temperature, 70 °C; temperature ramp, 10 °C/min; final temperature, 290 °C. Sample solution was filtered through a 0.22 μm PTFE syringe filter into a 2 mL vial and diethyl ether was added to fill the vial.

Abstraction Efficiency Calculation

To estimate the efficiency of hydrogen abstraction, we assumed that each productive abstraction event leads to a single fragmentation of the polymer chain. The abstraction efficiency (E) was defined as:

$$\mathrm{E}_{abs} = \frac{n}{n_{th}}\ x\ 100\ (eq. S1.1)$$

where n is the experimentally determined number of fragmentations events per chain and n_{th} is the theoretical maximum number of abstraction events per chain if all radicals generated per chain, from hydroperoxide decomposition, were to abstract a hydrogen atom and induce fragmentation.

The experimental number of abstraction events per chain, n, was obtained from the change in peak molecular weight (M_p) measured by GPC:

$$\frac{M_{\mathrm{p,0}}}{M_{\mathrm{p,t}}} = n + 1 \rightarrow n = \frac{M_{\mathrm{p,0}}}{M_{\mathrm{p,t}}} - 1\ (eq. S1.2)$$

where $M_{p,0}$ is the peak molecular weight at time 0 and $M_{p,t}$ is the peak molecular weight at reaction time, both determined by GPC. We selected M_p rather than M_n because M_p reflects the most abundant chain population and could therefore provide more representative and robust metrics for calculating the number of fragmentation events. The longer chains contain more abstractable C-H bonds and therefore statistically should be more likely to undergo H-abstraction and fragmentation.

GPC analysis representative example

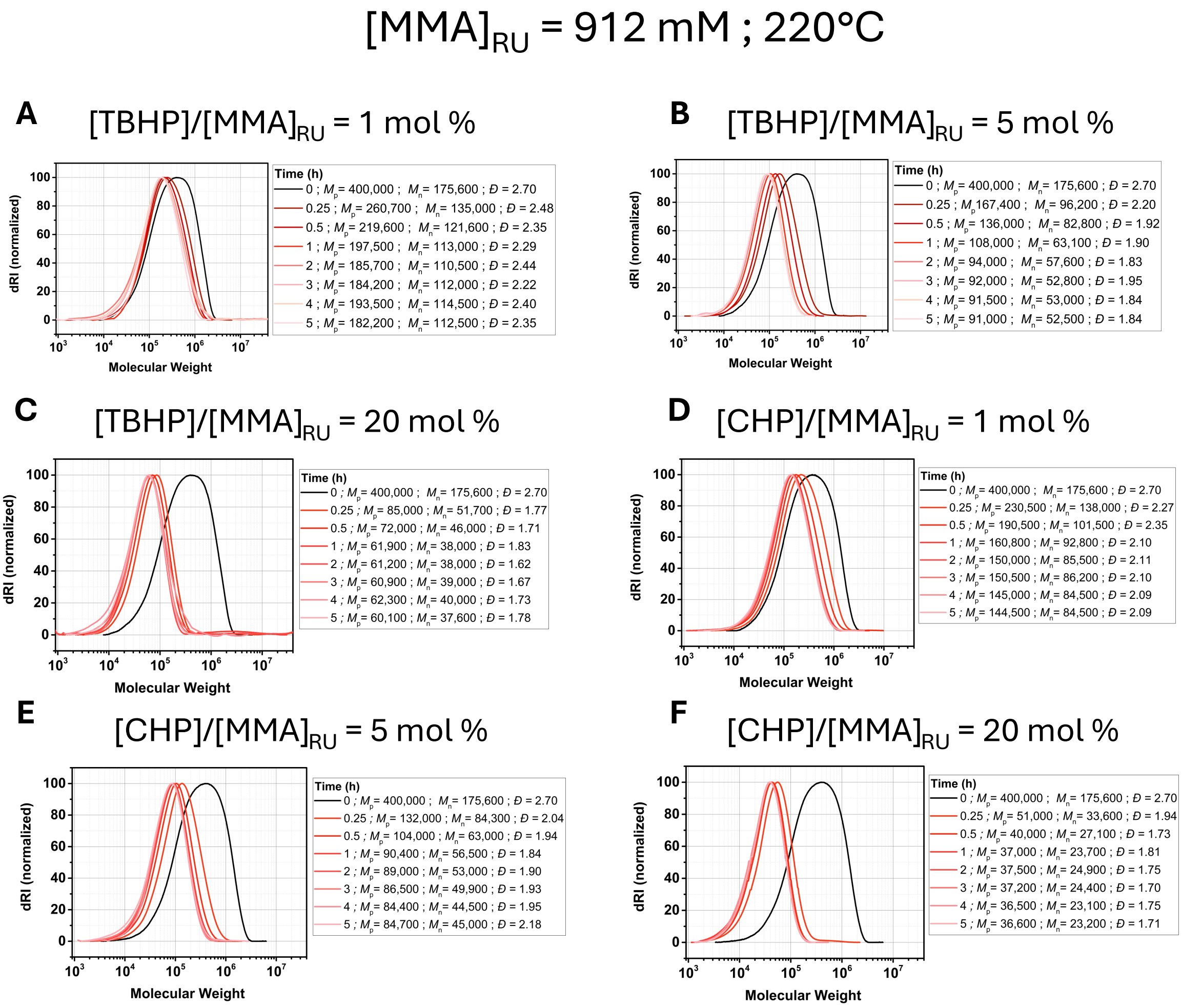

Figure S1. Representative GPC analysis of the reaction at 220°C with different $[HP]/[MMA]_{RU}$ (mol %).

Equilibrium monomer concentration and ceiling temperature

$$T_c = \frac{\boldsymbol{\Delta} H}{(\boldsymbol{\Delta} S^0 + R ln[M]_0)} \quad (eq. S2)$$

$$ln[M]_{eq} = \frac{\Delta H}{RT} - \frac{\Delta S^0}{R} \quad (eq. S3)$$

Polymer Synthesis

Procedure for PBMA, PPhMA and PBzMA synthesis

A typical procedure for the preparation of PBMA is described below. PBzMA and PPhMA were synthesized under identical conditions, i.e., the same molar ratios of reagents and overall monomer concentration, as well as the same reaction temperature and time. Butyl methacrylate (4.8 mL, 4.26 g, 30.0 mmol) was dissolved in toluene (10 mL, 32% v/v) in a Schlenk flask. AIBN (4.0 mg, 26.0 μmol, 0.085 mol% relative to the monomer) and DMF (93 μL, 1.2 mmol, 4 mol% relative to the monomer), used as an internal standard, were then introduced. The flask was equipped with a magnetic stir bar, sealed with a septum, and purged with argon for 20 min to remove dissolved oxygen. Polymerization was performed in a preheated oil bath at 70 °C and allowed to proceed overnight. Monomer conversion was evaluated by comparing the integral of a vinylic proton signal with that of the internal standard before and after the reaction. The polymerization was terminated by removing the reaction vessel from the oil bath and exposing it to air. The crude polymer was isolated by precipitation into cold methanol at least twice and subsequently dried under reduced pressure prior to use.

Polymer	M_n (kDa)	M_p (kDa)	$Đ$
**Poly (butyl methacrylate)**	85.7	227	1.94
**Poly (Benzyl methacrylate)**	96.5	203	2.65
**Poly (Phenyl methacrylate)**	149	210	2.41

General depolymerization procedure

PMMA stock solution 150 mg/mL was prepared in diphenyl ether. This solvent has a high boiling point (260°C) and no abstractable hydrogen atoms to avoid interference with the depolymerization process.

For a typical depolymerization performed at $[MMA]_{RU}$= 912 mM, 10 mol % [TBHP]/$[MMA]_{RU}$. 3.04 mL (456 mg of PMMA) of the stock solution was added in a 2-dram pressure release vial, TBHP (41 mg, 0.456 mmol, 59 mg/ 63.1 μL of a solution 70 % w/w) was added to the vial. The reaction was diluted to 5 mL with diphenyl ether. The solution was degassed for 30 min by sparging argon. The reaction was initiated by placing the vial in an oil bath at the desired temperature. Kinetic samples were retrieved using a degassed needle to monitor the kinetics at 0.25, 0.5, 1, 2, 3, 4, 5 h to record ^{1}H NMR and GPC.

The reactions at 170°C were performed with [MMA]RU = 320 mM to operate below $[MMA]_{eq}$. At 200 and 220°C, $[MMA]_{RU}$ was 912 mM at or below the $[MMA]_{eq}$ = 912 mM at 200°C, and 1.7 M at 220°C.

Quantification of depolymerization

For PMMA and the other polymethacrylates, depolymerization conversion was quantified by ^{1}H NMR using the same deconvolution protocol described below. The resonances of monomer, polymer, and consumed monomer in the 3.4–4.0 ppm region were deconvoluted to obtain the percentage of each species.

For PPhMA, deconvolution of the 3.4–4.0 ppm region was not possible because the monomer lacks isolated aliphatic ester resonances, so monomer concentration was determined by an external standard method using ethylene carbonate. In a typical analysis, an aliquot of the reaction mixture (10 μL) was mixed with an ethylene carbonate solution in $CDCl_3$ (10 μL, 15 mM) and diluted to 700 μL total volume with $CDCl_3$. Monomer concentration was obtained from the integral of the PPhMA vinyl proton signal (I_{vinyl}, n_{vinyl} = 2 H) and the ethylene carbonate methylene signal (I_{std}, n_{std} = 4 H) according to

$$[PhMA] = [EC]_{std} \times (I_{vinyl} / n_{vinyl}) / (I_{std} / n_{std})$$

where $[EC]_{std}$ is the known concentration of ethylene carbonate in the NMR tube. The depolymerization conversion was then calculated as

$$X = [PhMA]/[PhMA]_{RU,0}$$

with $[PhMA]_{RU,0}$ the initial PhMA repeating units concentration.

Reaction of MMA and 10 mol % t-butyl hydroperoxide at 220°C below $[M]_{eq}$

MMA (456.5 mg, 486 μL, 4.56 mmol) and TBHP (41.1 mg, 58.7 mg of the 70 % w/w, 63.8 μL of the solution 70 % w/w, 0.456 mmol) were added to a pressure-release vial, and the volume was filled to 5 mL with diphenyl ether. The solution was degassed by sparging argon for 30 min. The reaction was started by putting the vial on a heating holder at 220°C. The reaction was sampled at 0.25, 0.5, 1, 2, 3, 4, 5h to record ^{1}H NMR and GPC.

Reaction of MMA and 10 mol % cumene hydroperoxide at 220°C below $[M]_{eq}$

MMA (456.5 mg, 486 μL, 4.56 mmol) and CHP (69.4 mg, 86.73 mg of the solution 80 % w/w, 84.2 μL of the solution 80 % w/w, 0.456 mmol) were added to a pressure-release vial, and the volume was filled to 5 mL with diphenyl ether. The solution was degassed by sparging argon for 30 min. The reaction was started by putting the vial on a heating holder at 220°C. The reaction was sampled at 0.25, 0.5, 1, 2, 3, 4, 5h to record ^{1}H NMR and GPC.

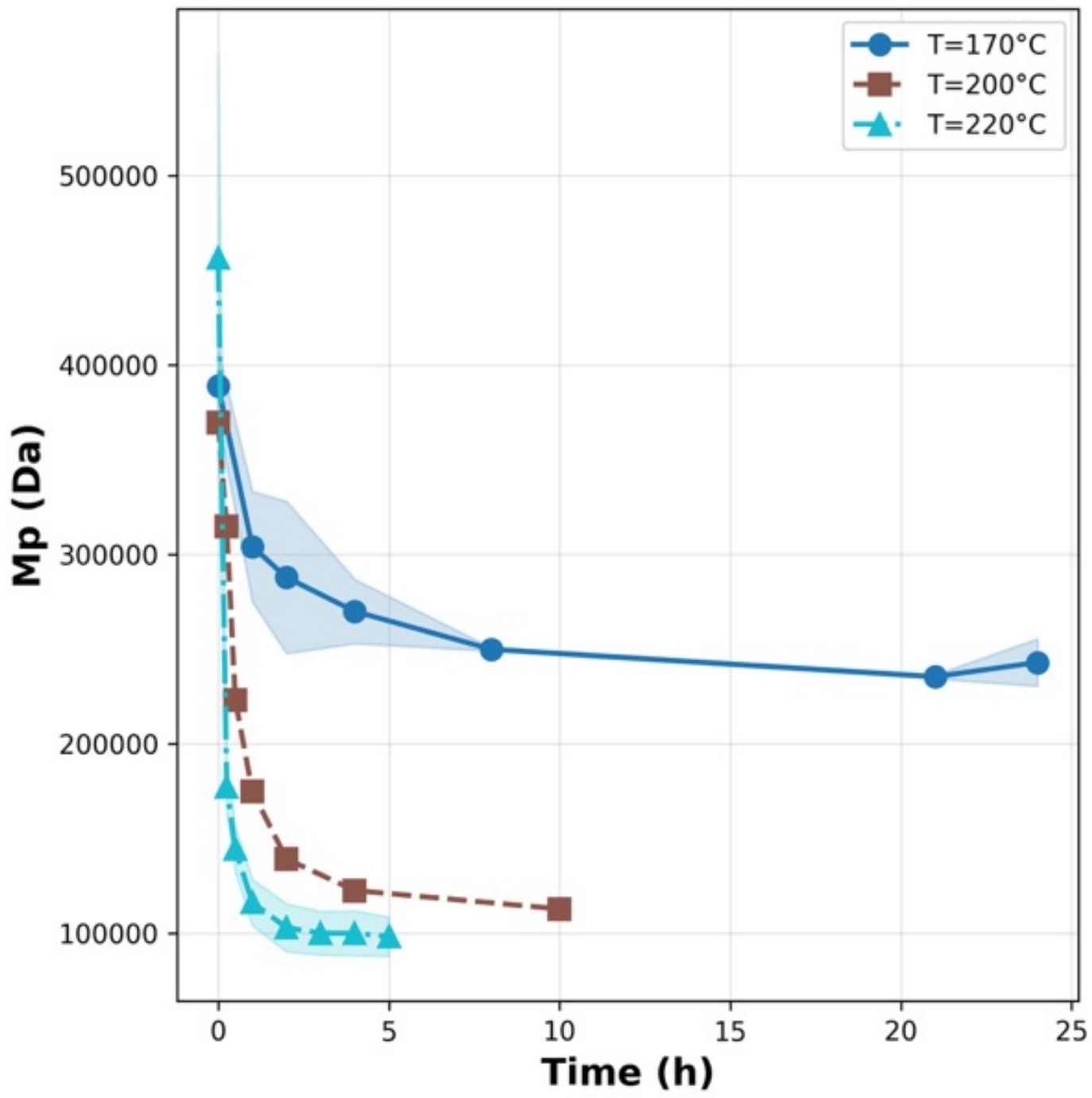

Figure S2. Decrease of peak molecular weight (M_p) with time at $[TBHP]/[MMA]_{RU}$ = 5 mol % (RU = Repeating Unit). The shaded area represents ± one standard deviation from experiments performed in triplicate (n = 3).

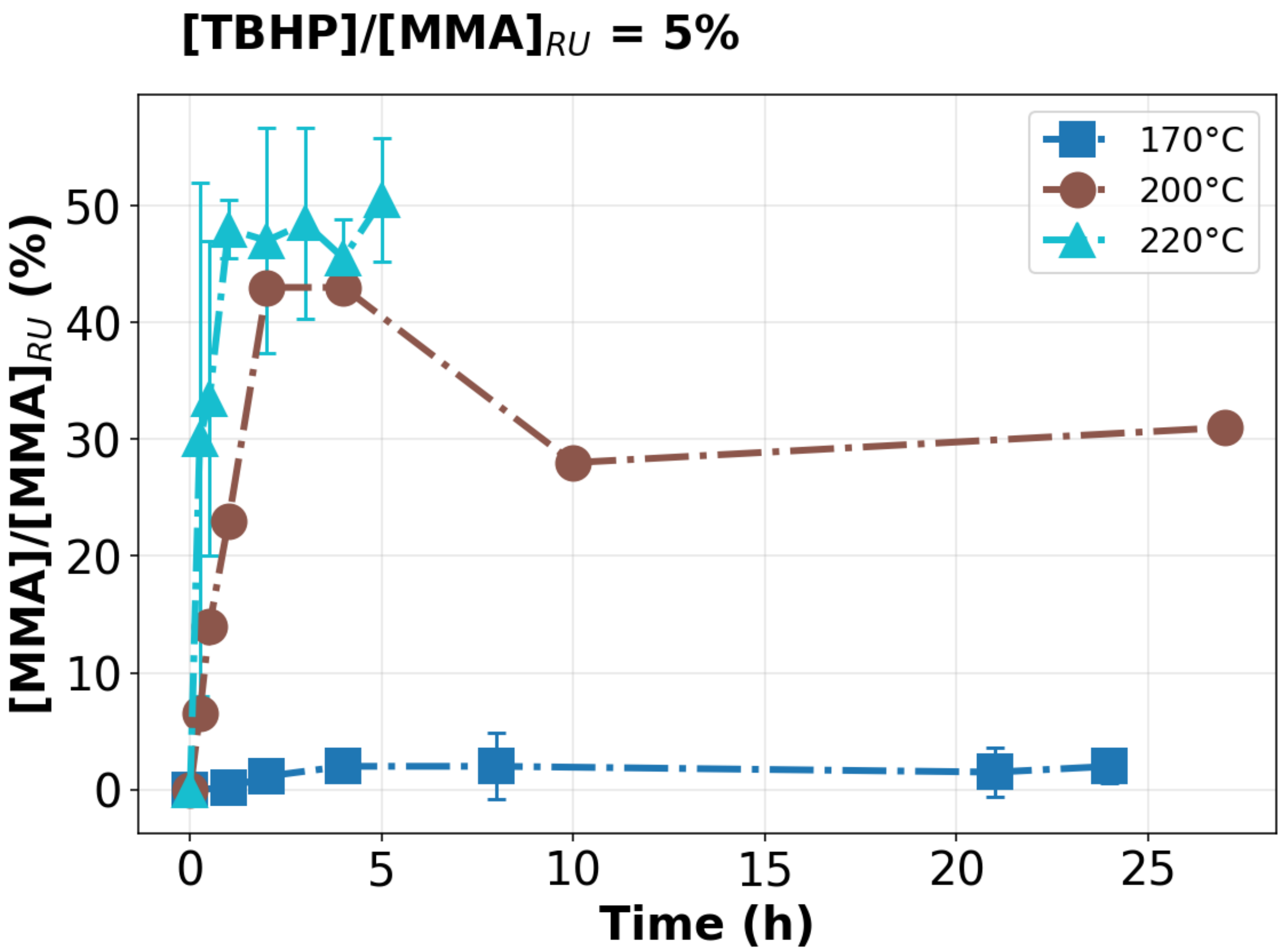

Figure S3. Kinetics of depolymerization at 170-220°C and $[TBHP]/[MMA]_{RU}$ = 5 mol %.

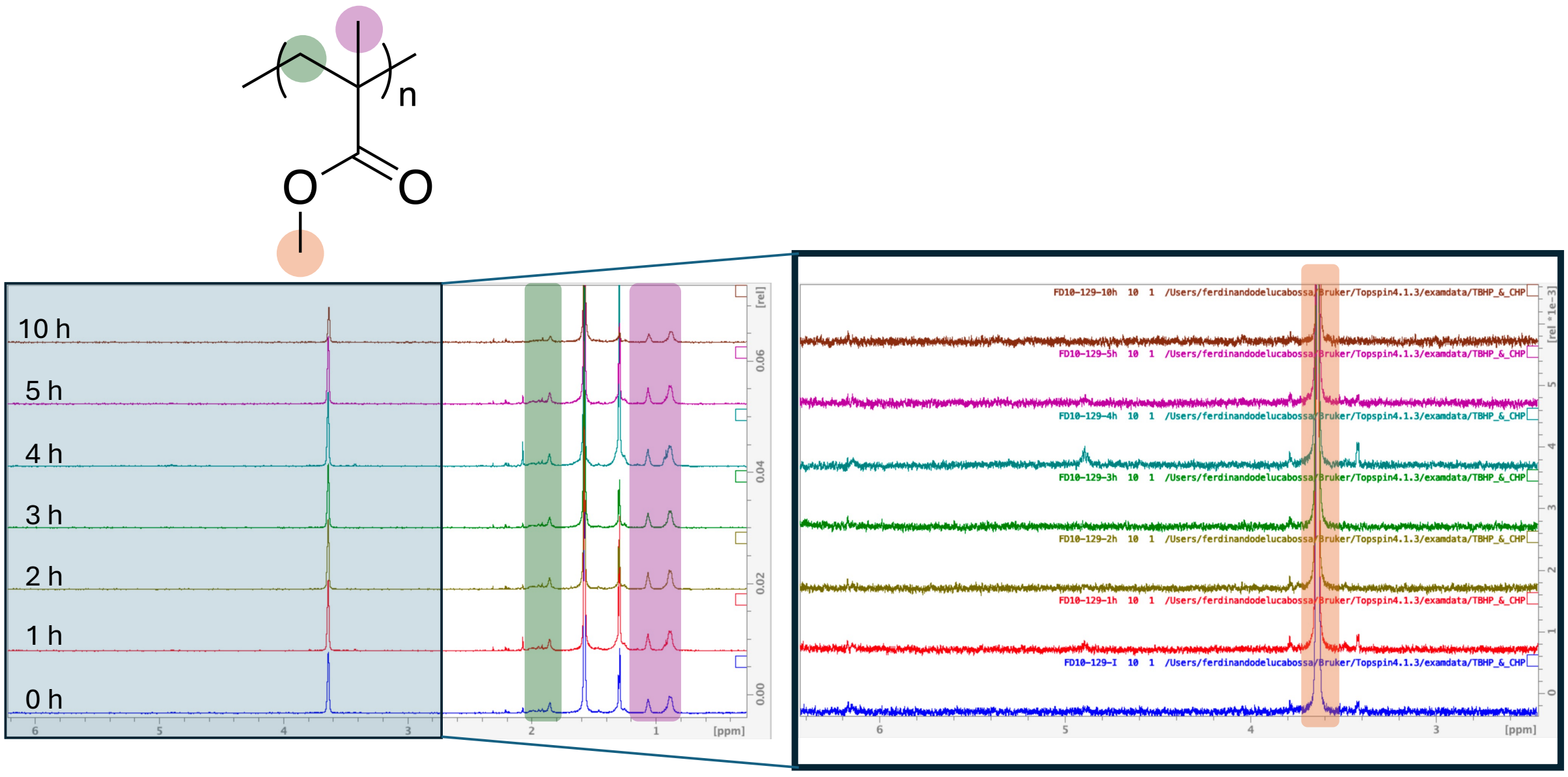

Figure S4. ^{1}H NMR spectra recorded during kinetic study of depolymerization at 170°C and [TBHP]/$[MMA]_{RU}$ = 10 mol %, $[MMA]_{RU}$ = 50 mM.

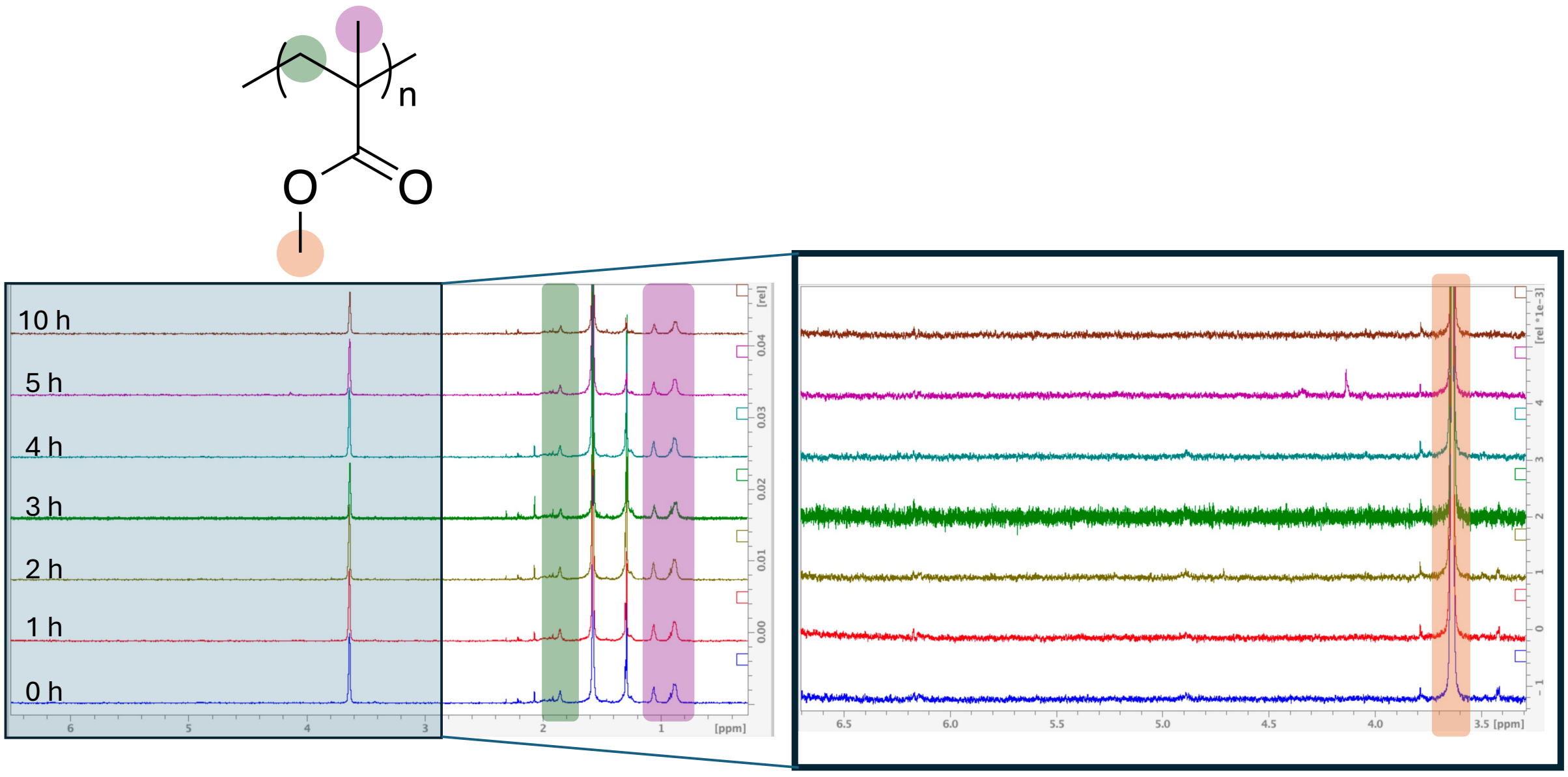

Figure S5. ^{1}H NMR spectra recorded during kinetic study of depolymerization at 170°C and [TBHP]/$[MMA]_{RU}$ = 5 mol %, $[MMA]_{RU}$ = 50 mM.

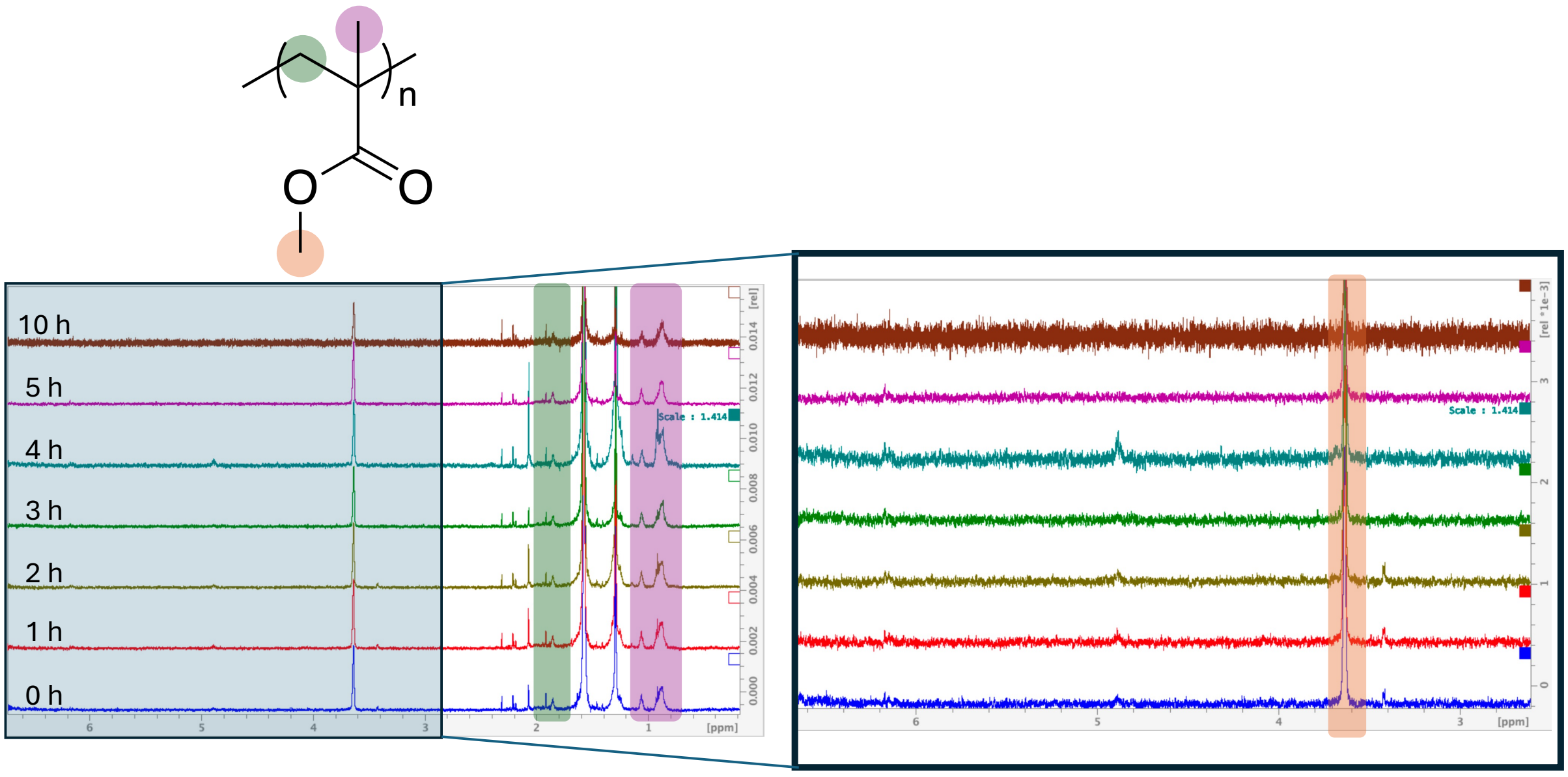

Figure S6. ^{1}H NMR spectra recorded during kinetic study of depolymerization at 170°C and [TBHP]/$[MMA]_{RU}$ = 10 mol %, $[MMA]_{RU}$ = 10 mM.

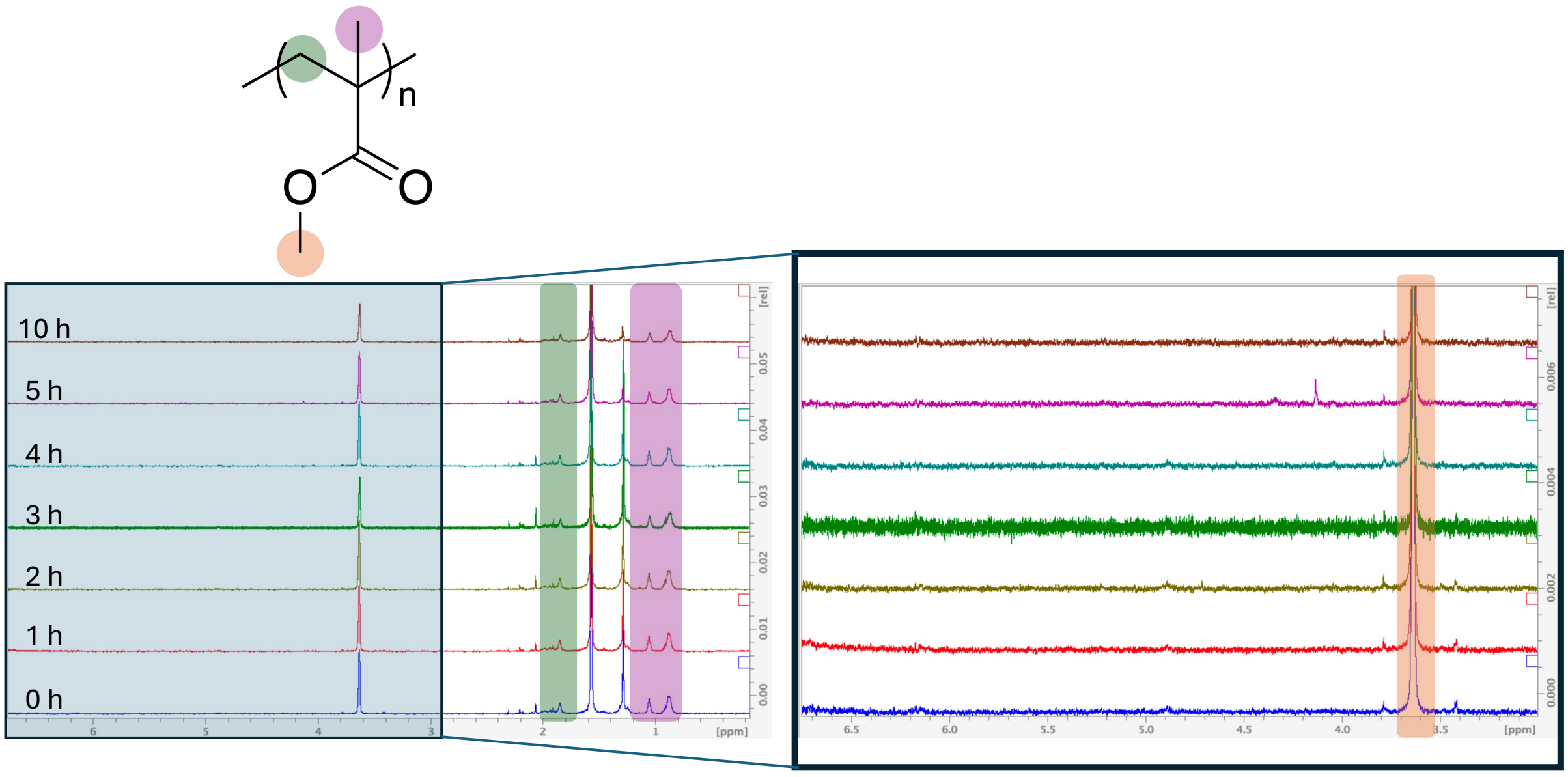

Figure S7. ^{1}H NMR spectra recorded during kinetic study of depolymerization at 170°C and [TBHP]/[MMA]$_{RU}$ = 5 mol %, [MMA]$_{RU}$ = 10 mM.

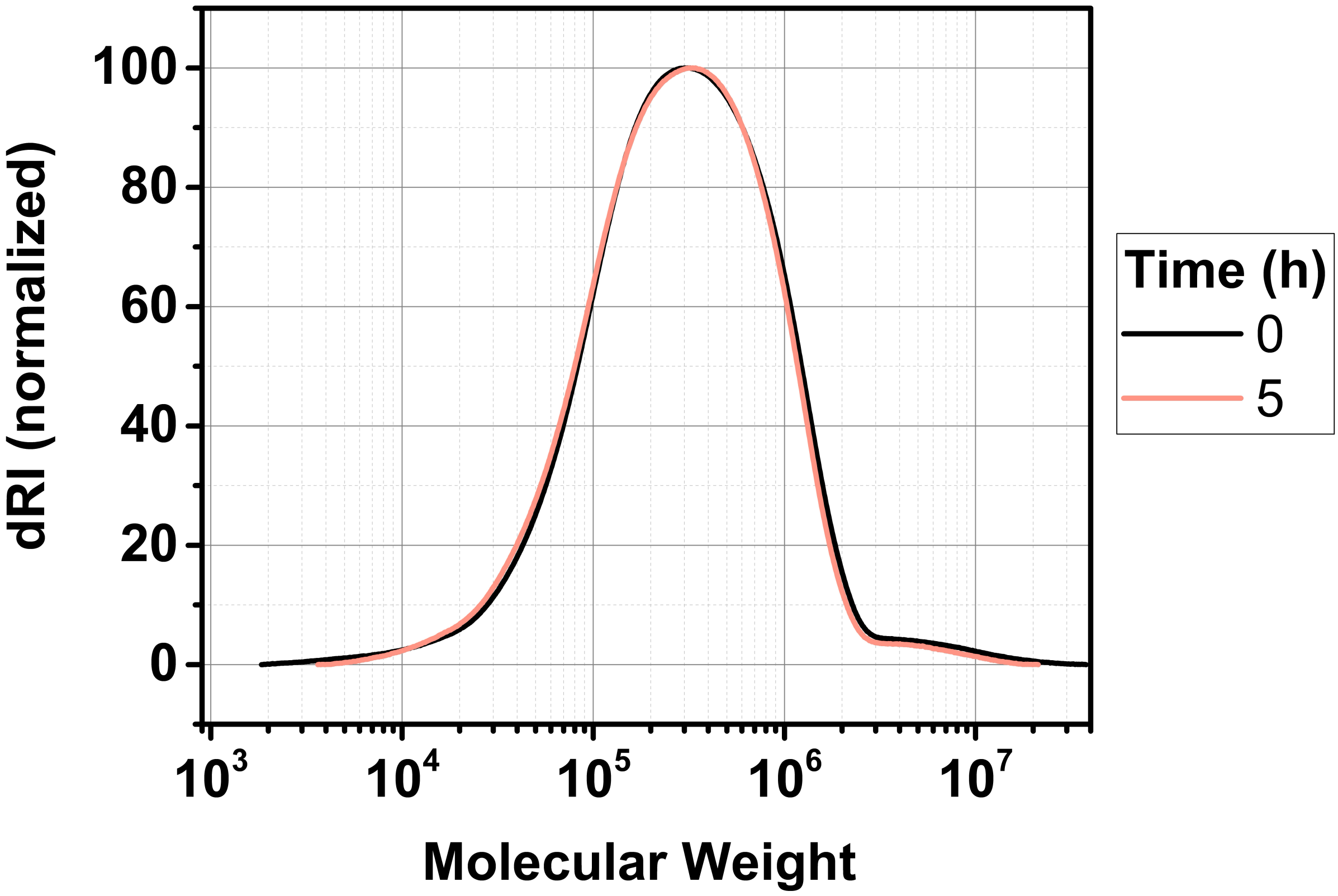

Figure S8. PMMA MW evolution ($[MMA]_{RU}$ = 912 mM) in diphenyl ether at 220°C after 5h.

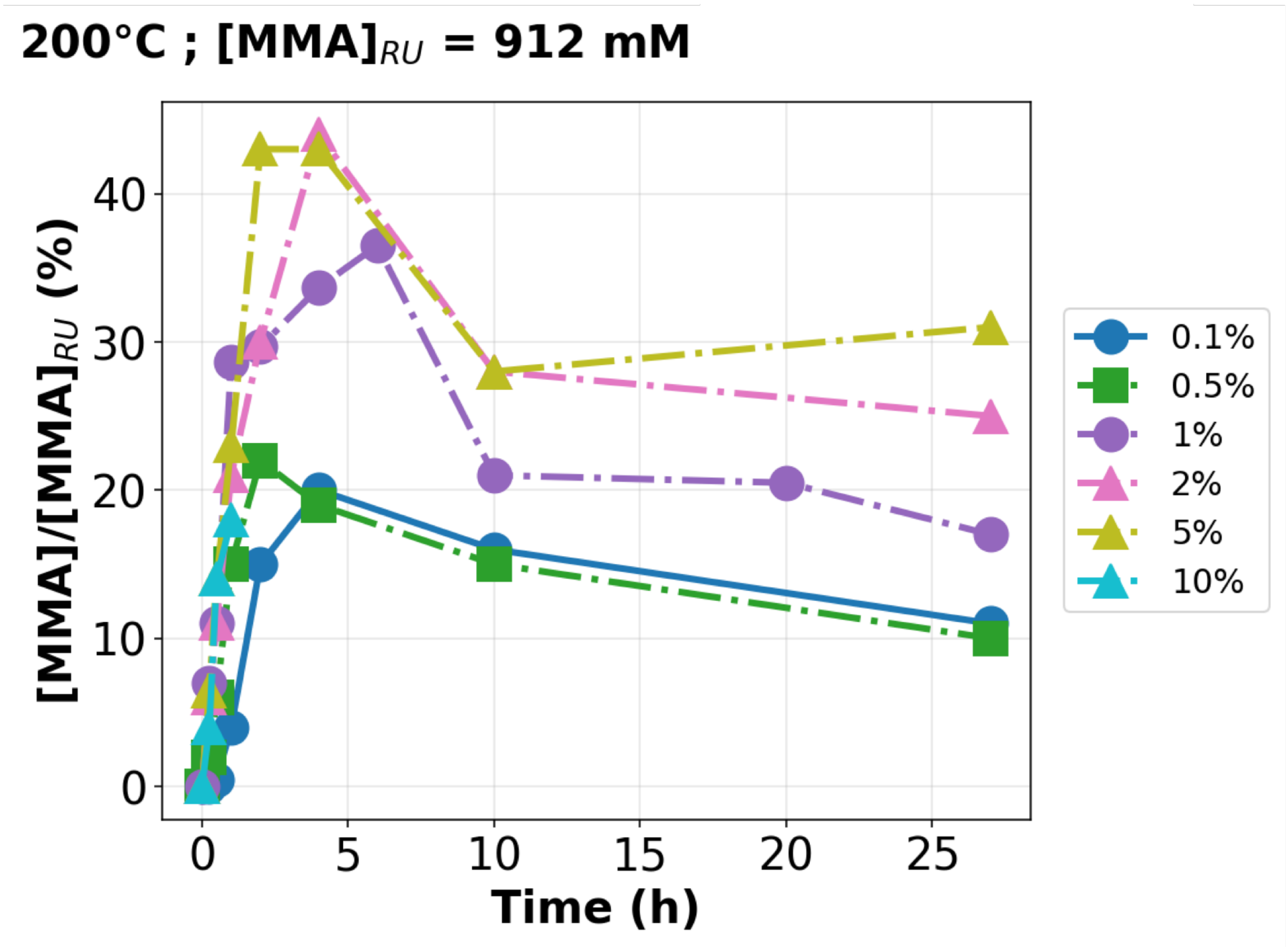

Figure S9. Kinetics of depolymerization at 200°C and different molar ratios [TBHP]/[MMA]$_{RU}$ (0.1, 0.5, 1, 2, 5, 10, mol %).

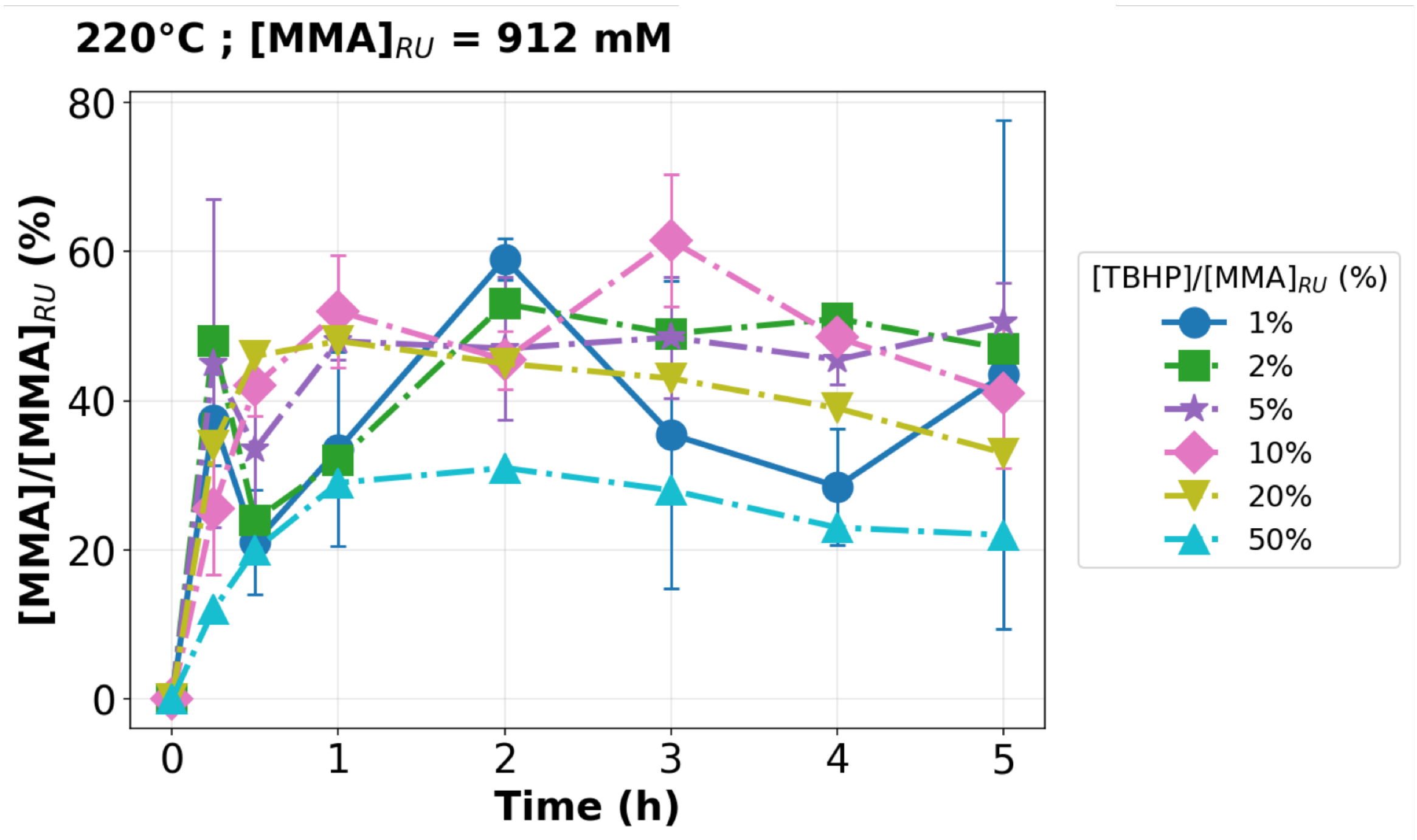

Figure S10. Kinetics of depolymerization at 220°C and different molar ratios [TBHP]/[MMA]$_{RU}$ (1, 2, 5, 10, 20, 50 mol %).

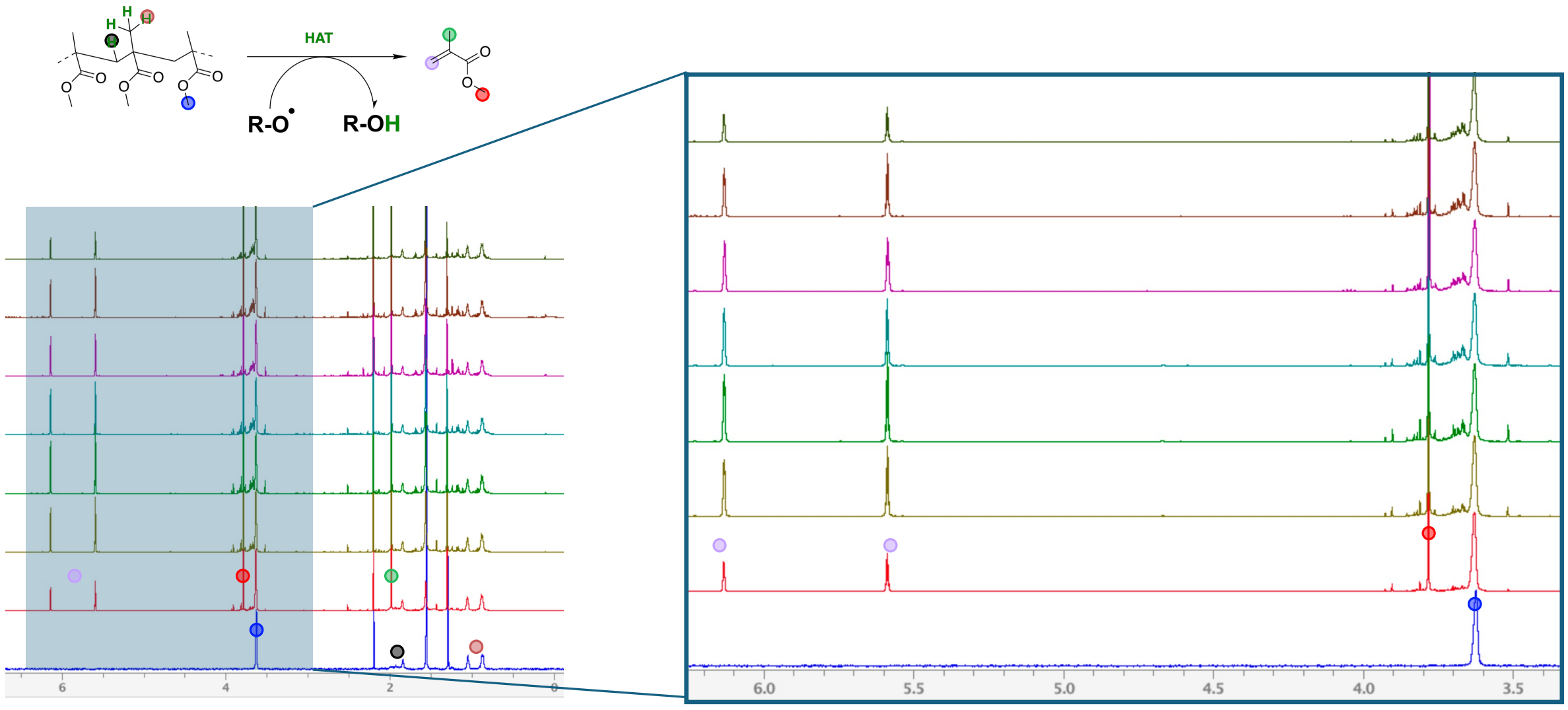

Figure S11. Example of kinetic analysis of depolymerization at 220°C with $[TBHP]/[MMA]_{RU}$ = 20 mol % and $[MMA]_{RU}$ = 912 mM. The formation of additional peaks in the region 3.4-4.0 ppm is visible. ^1H NMR was taken in $CDCl_3$ at 0, 0.25, 0.5, 1, 2, 3, 4, 5 h

Deconvolution

Deconvolution of the ^{1}H NMR spectra performed using the built-in deconvolution function in Bruker TopSpin software. Signals in the 3.4-4.0 ppm region were deconvoluted to quantify the relative contributions from polymer (3.63 ppm), monomer (3.78 ppm), and consumed monomer, the latter determined by difference. The authors selected the region to be deconvoluted (3.4-4.0 ppm), and the software identified the peaks and provided a report containing the area of each peak. The relative percentage of each species was calculated as the ratio of the corresponding area to the sum of all areas.

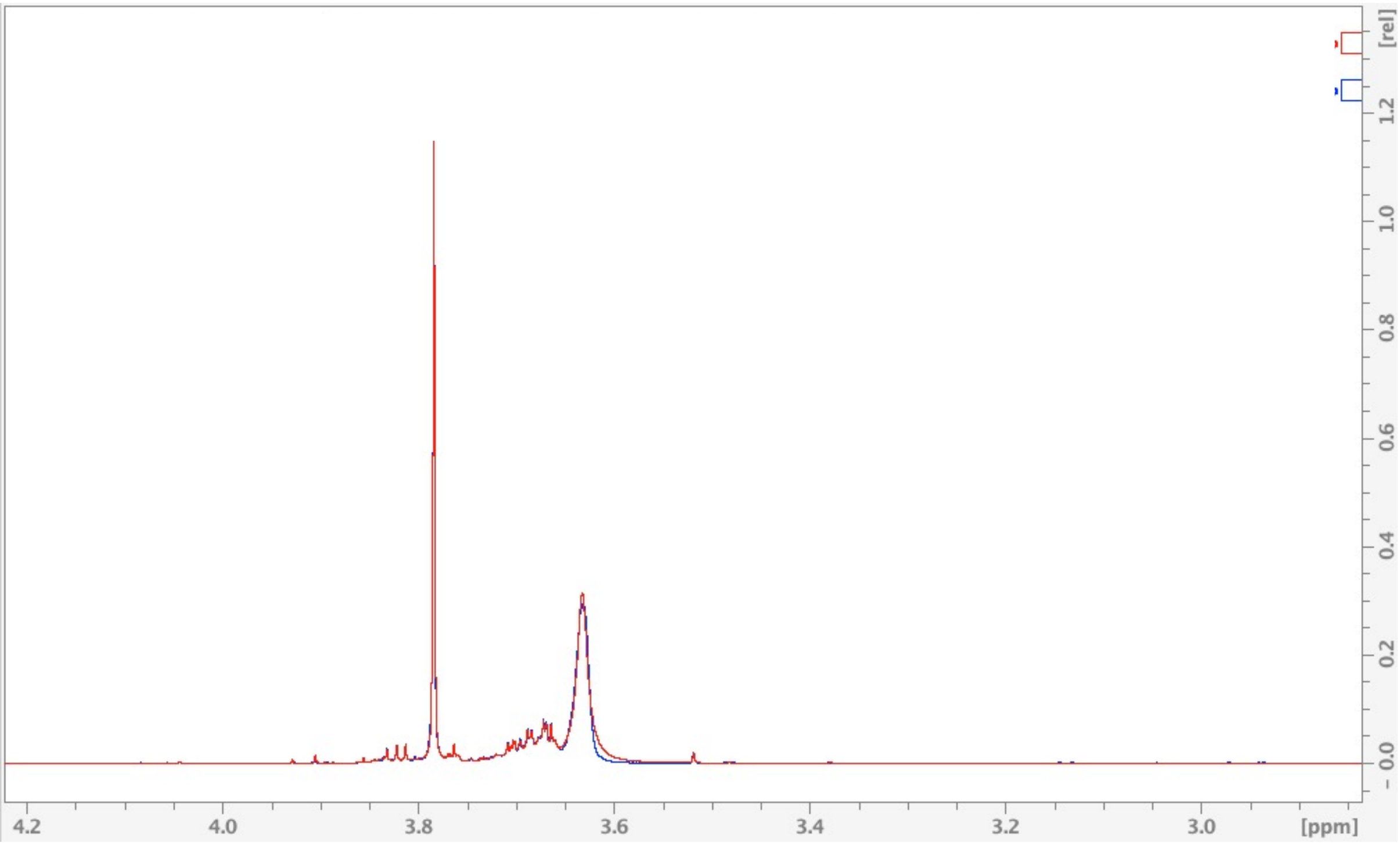

Figure S12. Deconvolution example of the depolymerization reaction mixture after 5h at 220°C using [TBHP]/[MMA]$_{RU}$ = 20 mol %. 50% PMMA, 23 % intact MMA, 27 % consumed MMA.

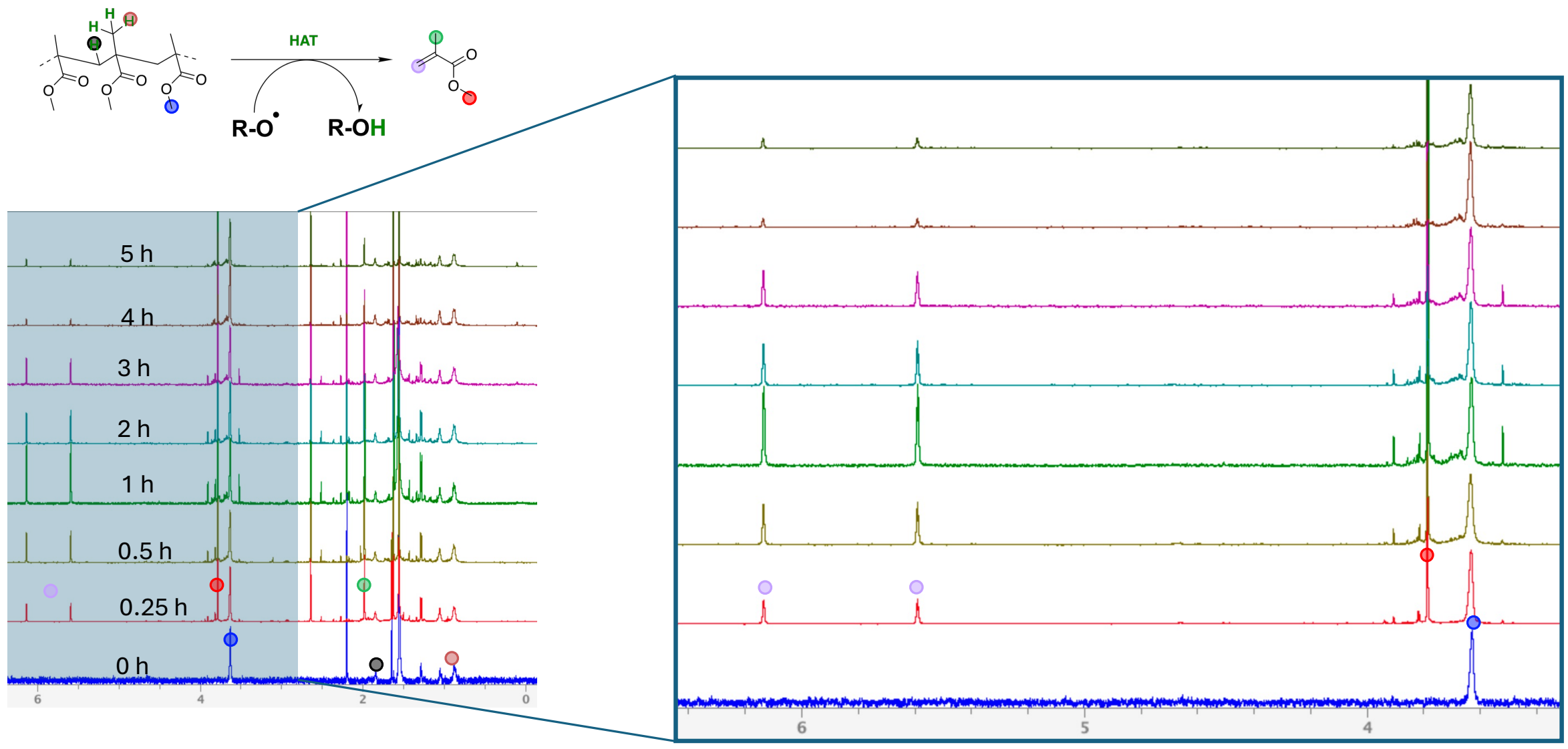

Figure S13. Example of kinetic analysis of depolymerization at 220°C with [CHP]/[MMA]$_{RU}$ = 20 mol % and [MMA]$_{RU}$ = 912 mM. The formation of additional resonance in the region between 3.4-4.0 ppm is visible. ^{1}H NMR was taken in $CDCl_3$ at 0, 0.25, 0.5, 1, 2, 3, 4, 5 h

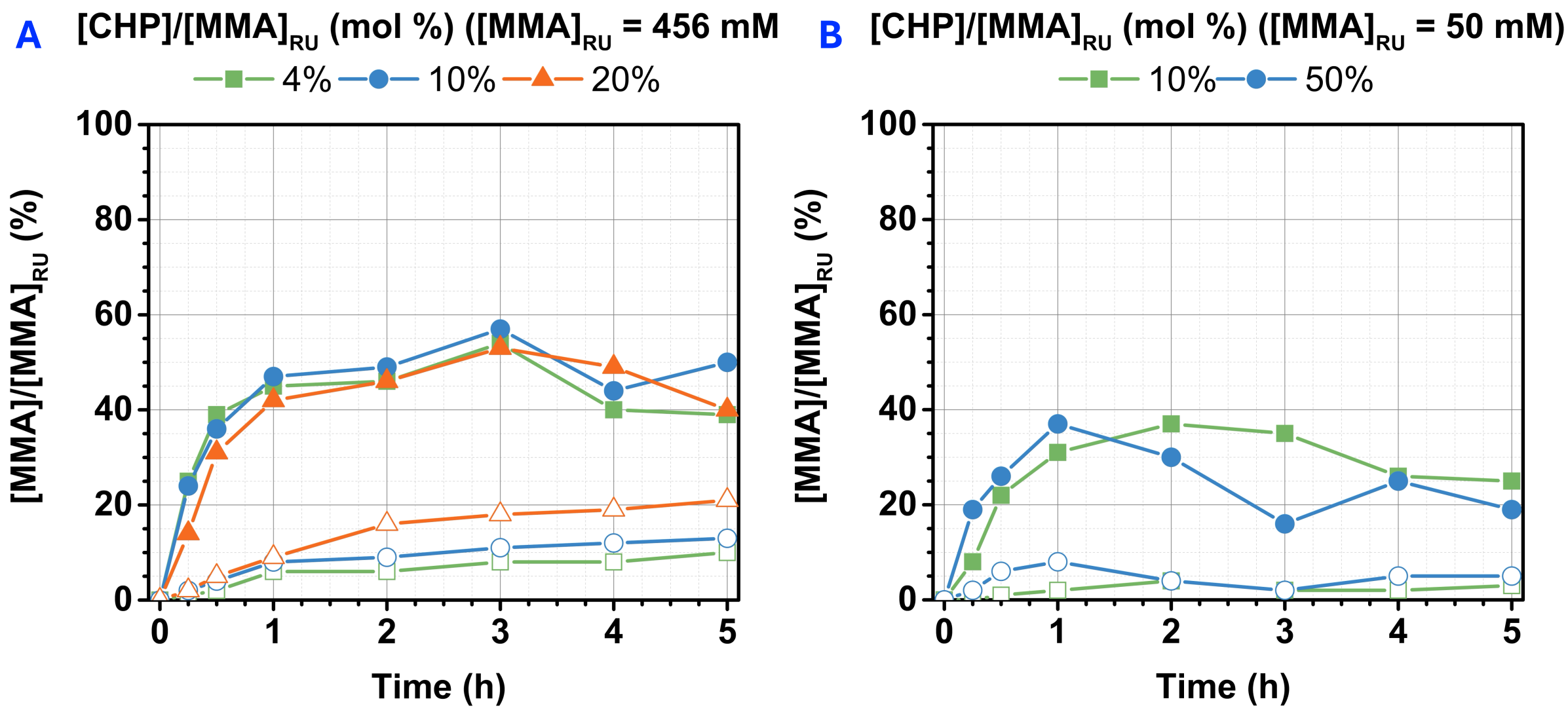

Figure S14. Depolymerization kinetics using CHP with different [RU] at 220°C.

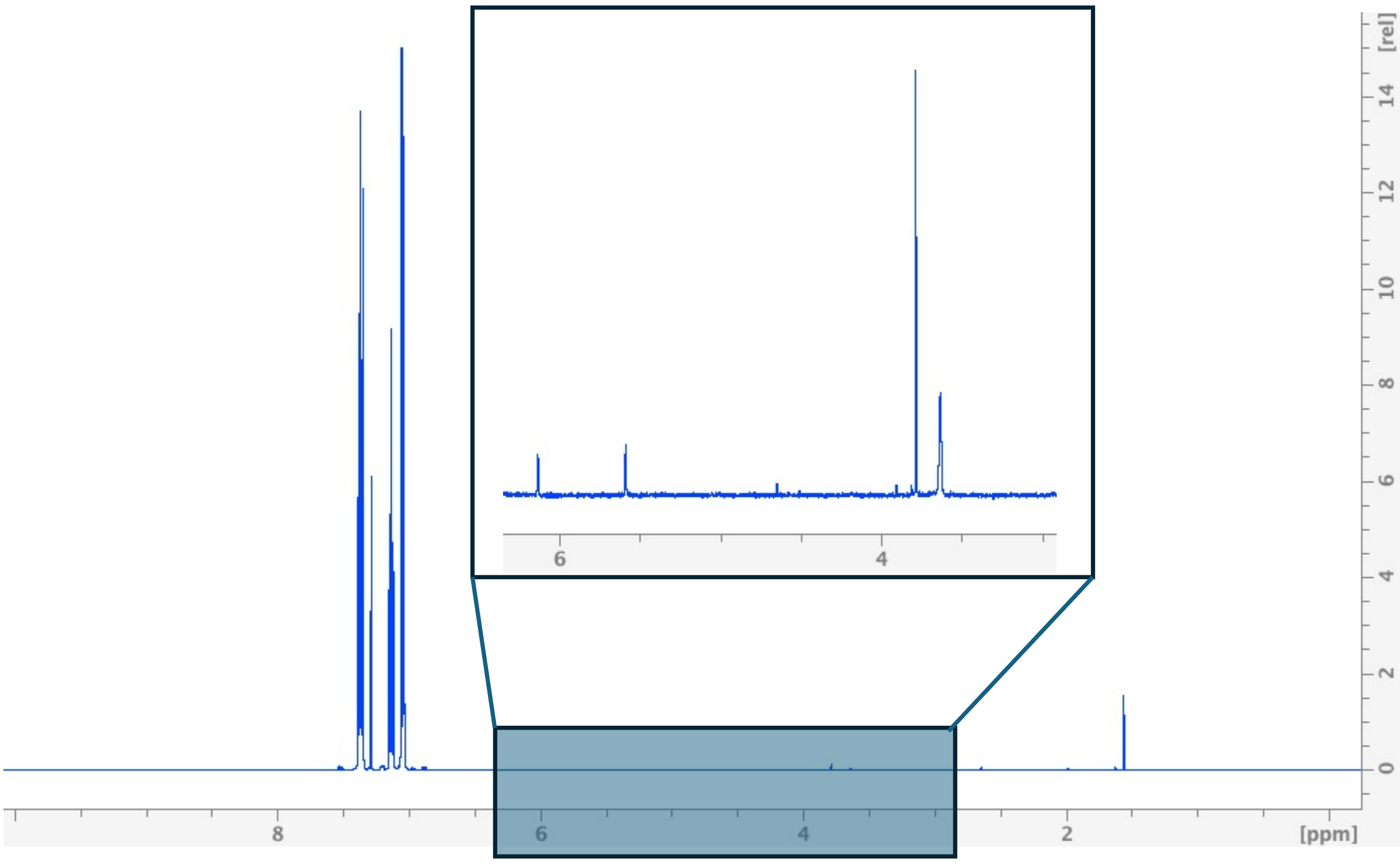

Figure S15. ^{1}H NMR spectrum of depolymerization at 220°C with $[CHP]/[MMA]_{RU}$ = 10 mol % and $[MMA]_{RU}$ = 50 mM.

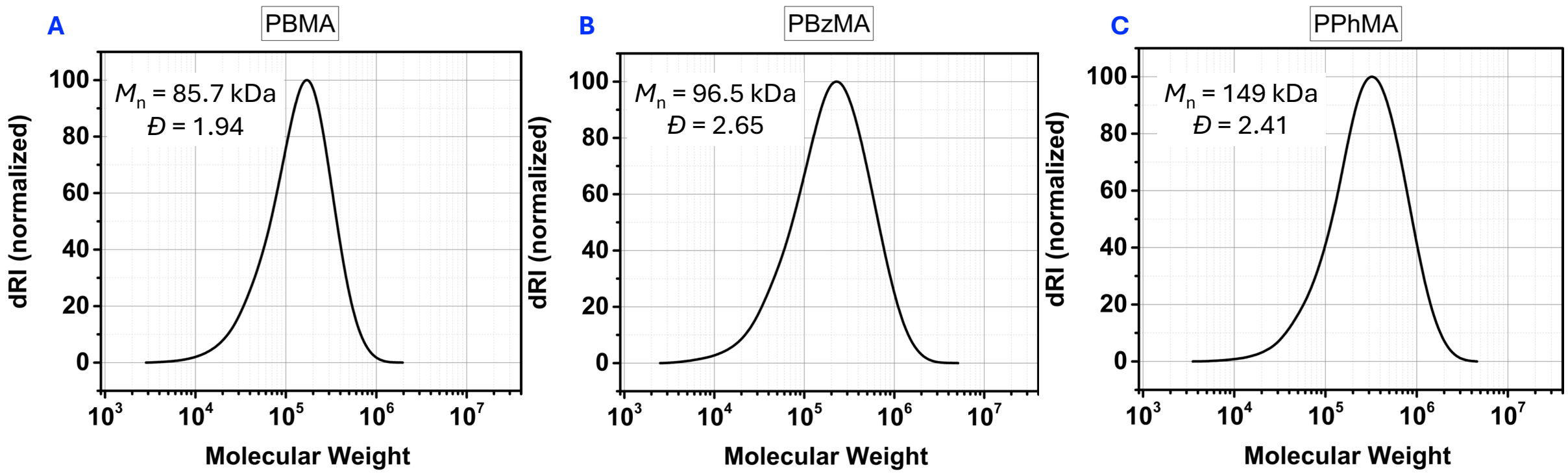

Figure S16. GPC of the polymethacrylates synthesized by FRP.

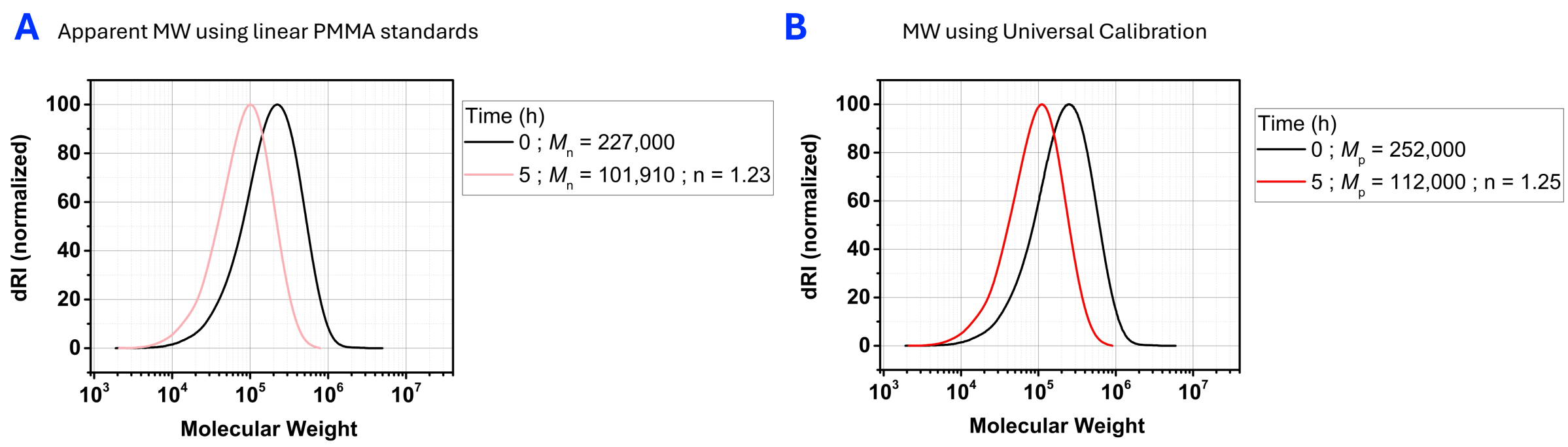

Figure S17. Comparison of the number of fragmentations calculated using linear PMMA standards and universal calibration. Mark-Houwink parameters used : K_{PMMA} = 7.56 x 10^{-5} dLg^{-1}, α_{PMMA} = 0.731, K_{PBMA} = 9.64 x 10^{-5} dLg^{-1}, α_{PBMA} = 0.696.[1]

[Plexiglas]$_{RU}$ = 912mM ; [TBHP]/[MMA]$_{RU}$ = 10 mol% ; 220°C ; Diphenyl ether

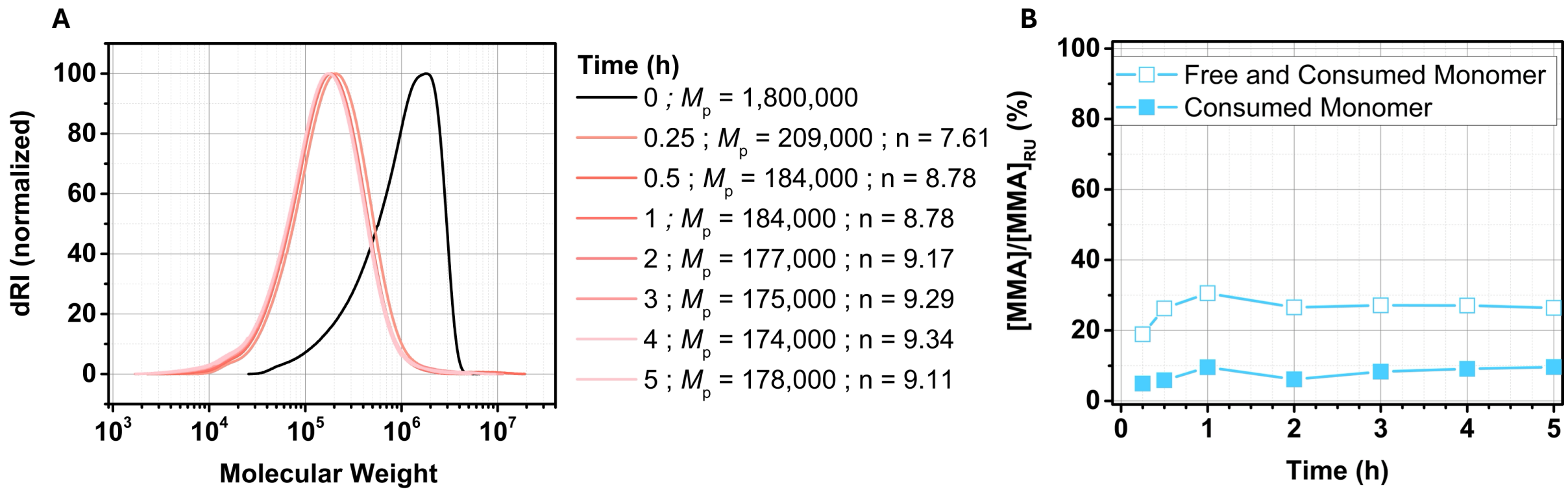

Figure S18. Results of the depolymerization reaction using a Plexiglas sheet.

Scheme S 1. Mechanism of decomposition of TBHP, and degradation of the corresponding alkoxyl radical.

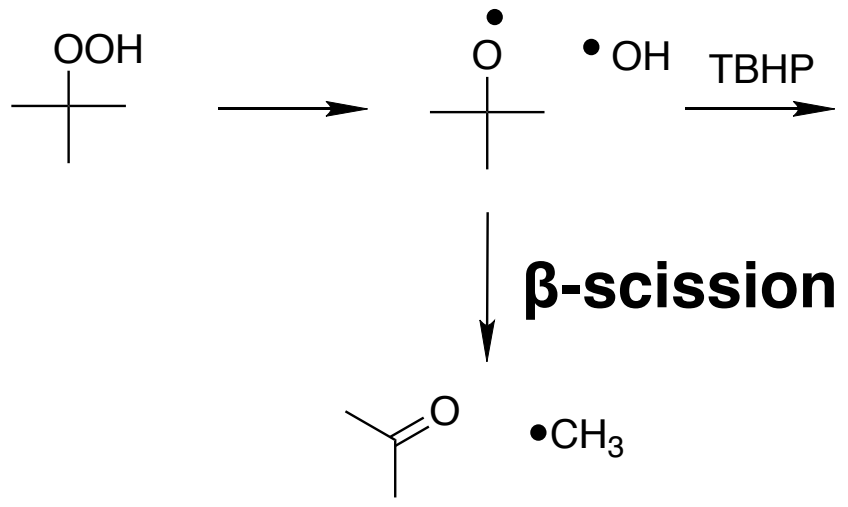

Scheme S 2. Mechanism of decomposition of CHP, and degradation of the corresponding alkoxyl radical.

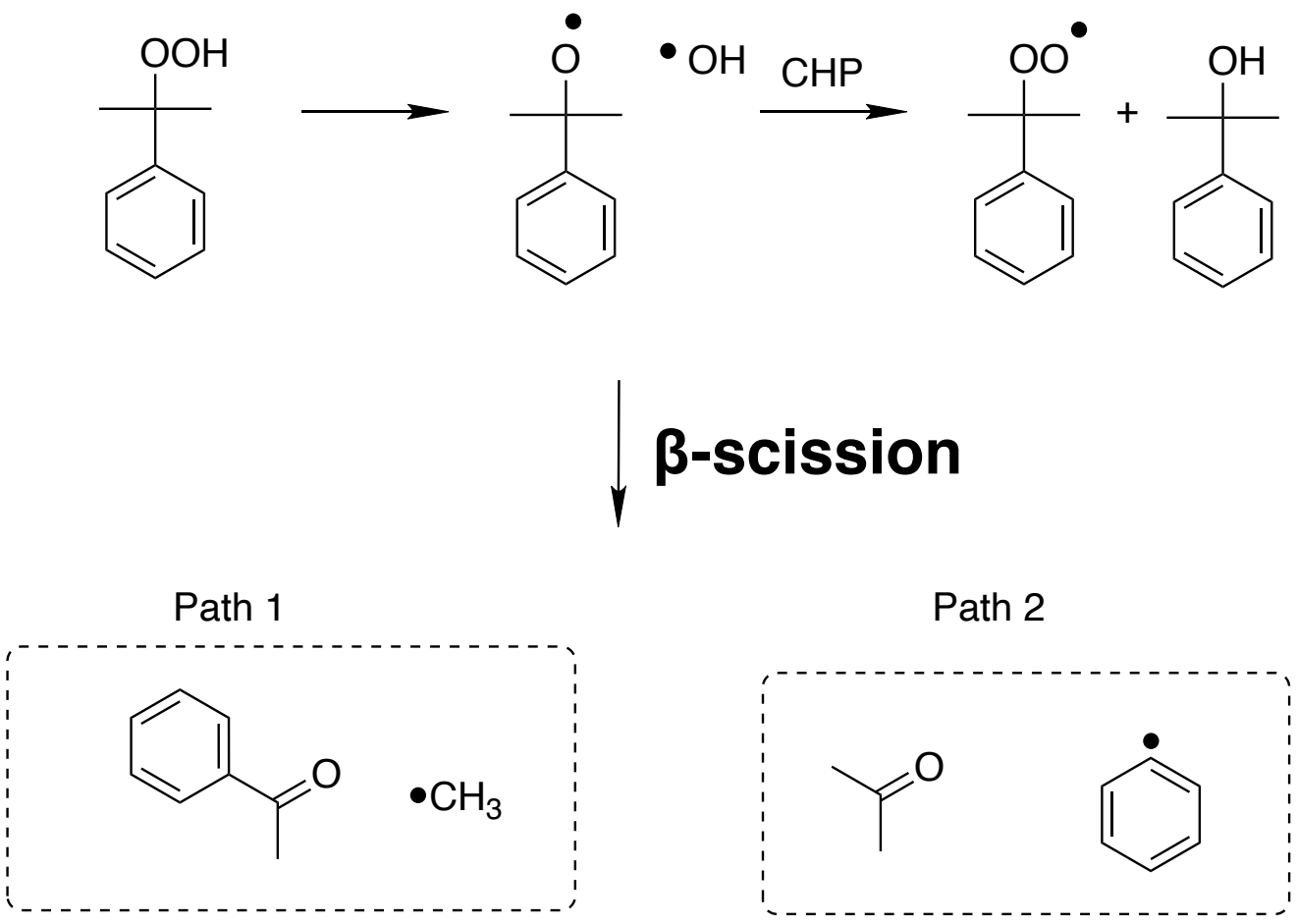

Discussion on methyl radical capability of abstracting H-atoms from polymethacrylates

The H-abstraction capability of a radical should correlate with the bond dissociation energy (BDE) of the bond formed. The BDE represents the thermodynamic driving force of the process. The kinetics of the abstraction follows a separate rule. The Marcus cross relation, developed by Mayer's group, approximates the rate constant of a hydrogen atom transfer (HAT) reaction.[2–6]

$$A - H + B \rightarrow A + H - B$$

$$k_{AH/B} = \sqrt{k_{AH/A} k_{BH/B} K_{eq_{AH/B}} f}$$

The rate constant of a generic abstraction reaction is the square root of three terms. These are the product of the two self-exchange rate constants ($k_{AH/A}$ and $k_{BH/B}$), the equilibrium constant of the reaction (K_{eq}, AH/B), and a factor f. The factor f is a correction term that accounts for the work required to bring the reactants together when the reaction is not thermoneutral.

C-H bonds are characterized by intrinsically slow self-exchange rate constants. This is a general feature of carbon-centered radicals: the reorganization energy required for hydrogen transfer between a C-H bond and the corresponding carbon radical is large, resulting in self-exchange rate constants that are many orders of magnitude lower than those of O-H or N-H bonds.[6] The contrast is striking when comparing two substrates of similar BDE: toluene (C-H BDE ~ 88 kcal mol^{-1}, $k_{\text{H self-exchage}}$ ~ 8 × 10^{-5} M^{-1} s^{-1}) and phenol (O-H BDE ~ 86 kcal mol^{-1}, $k_{\text{H self-exchage}}$ ~ 3 × 10^{5} M^{-1} s^{-1}).[7] Despite their nearly identical thermodynamic driving forces, the HAT rate from phenol exceeds that from toluene by approximately ten orders of magnitude, a purely kinetic effect governed by the self-exchange term in the Marcus cross relation.[6]

This same kinetic penalty applies to the abstraction of a hydrogen atom from PMMA by a methyl radical. The C-H BDE of methane (~100-105 kcal mol^{-1}) is close to the C-H BDE of the α-position of PMMA (~100-105 kcal mol^{-1}), so the reaction has little thermodynamic driving force. More importantly, both self-exchange rate constants, for the C-H bonds in PMMA and methane, are inherently low, following the same pattern established for all C-H/carbon-radical pairs.

The O–H BDEs in tert-butyl alcohol and tert-butyl hydroperoxide are ~106 kcal mol^{-1} and ~85 kcal mol^{-1}, respectively. A *t*-butyl peroxyl radical will therefore have no thermodynamic driving force to abstract a hydrogen from PMMA. The *t*-butoxyl radical shows a modest thermodynamic driving force which is comparable to that of the methyl radical for abstraction from PMMA. However, it benefits from a much faster O-H

self-exchange process, estimated to be for *t*-BuO· and *t*-BuOO· 3×10^4 M^{-1} s^{-1} and 5×10^2 M^{-1} s^{-1}, respectively.[4] As a result, HAT from PMMA by *t*-BuO· can be orders of magnitude faster than the analogous Me·, even at similar BDE differences.

In other words, a HAT process requires two conditions. The first is a thermodynamic driving force, set by the BDE difference between the bond broken and the bond formed. The second is a polarity match.[8] An electrophilic radical preferentially abstracts a nucleophilic H atom. A nucleophilic radical abstracts an electrophilic H atom more efficiently. The other combinations are disfavoured.

Experiments with MMA

Table S1. ^{1}H NMR results of MMA polymerization at 220°C, $[MMA]_{RU}$ = 912 mM, $[TBHP]/[MMA]_{RU}$ = 10%.

Time (h)	[Vinyl]/ [Methyl Ester]	Conversion (%)	Polymer detected (Y/N)
0	66	0	N
0.25	55	17	N
0.5	48	27	N
1	41	38	N
2	41	38	N
3	36	45	N
4	35	47	N
5	34	48	N

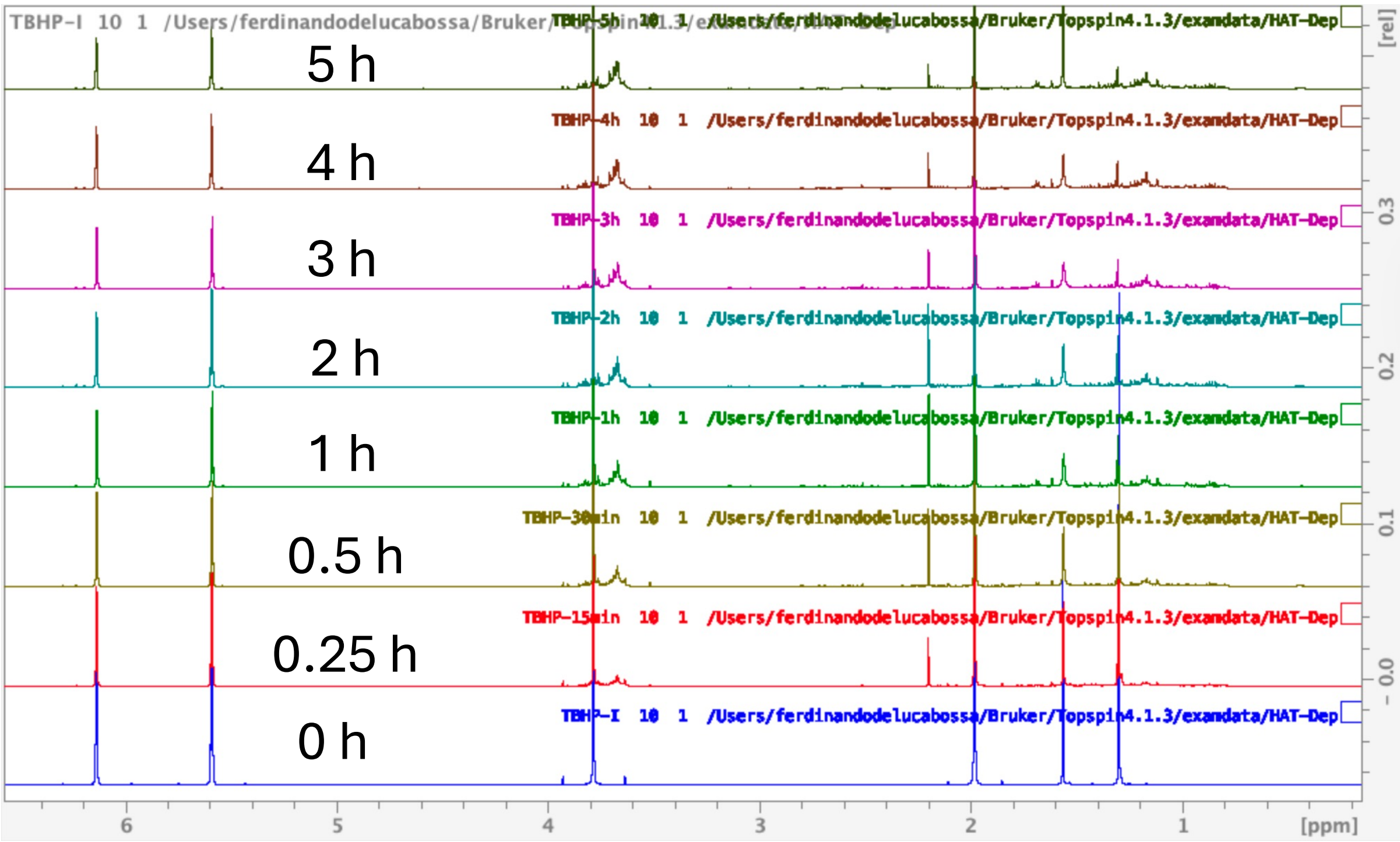

Figure S19. ^{1}H NMR spectra in $CDCl_3$ of MMA polymerization using 10 mol % [TBHP]/[MMA] at 220°, and [MMA] = 912 mM (time points at 0, 0.25, 0.5, 1, 2, 3, 4, 5 h).

Table S2. ^{1}H NMR results of MMA polymerization at 220°C, $[MMA]_{RU}$ = 912 mM, $[CHP]/[MMA]_{RU}$ = 10%.

Time (h)	[Vinyl]/ [Methyl Ester]	Conversion (%)	Polymer detected (Y/N)
0	67	0	N
0.25	47	30	N
0.5	42	37	N
1	40	40	N
2	38	43	N
3	35	48	N
4	36	46	N
5	35	48	N

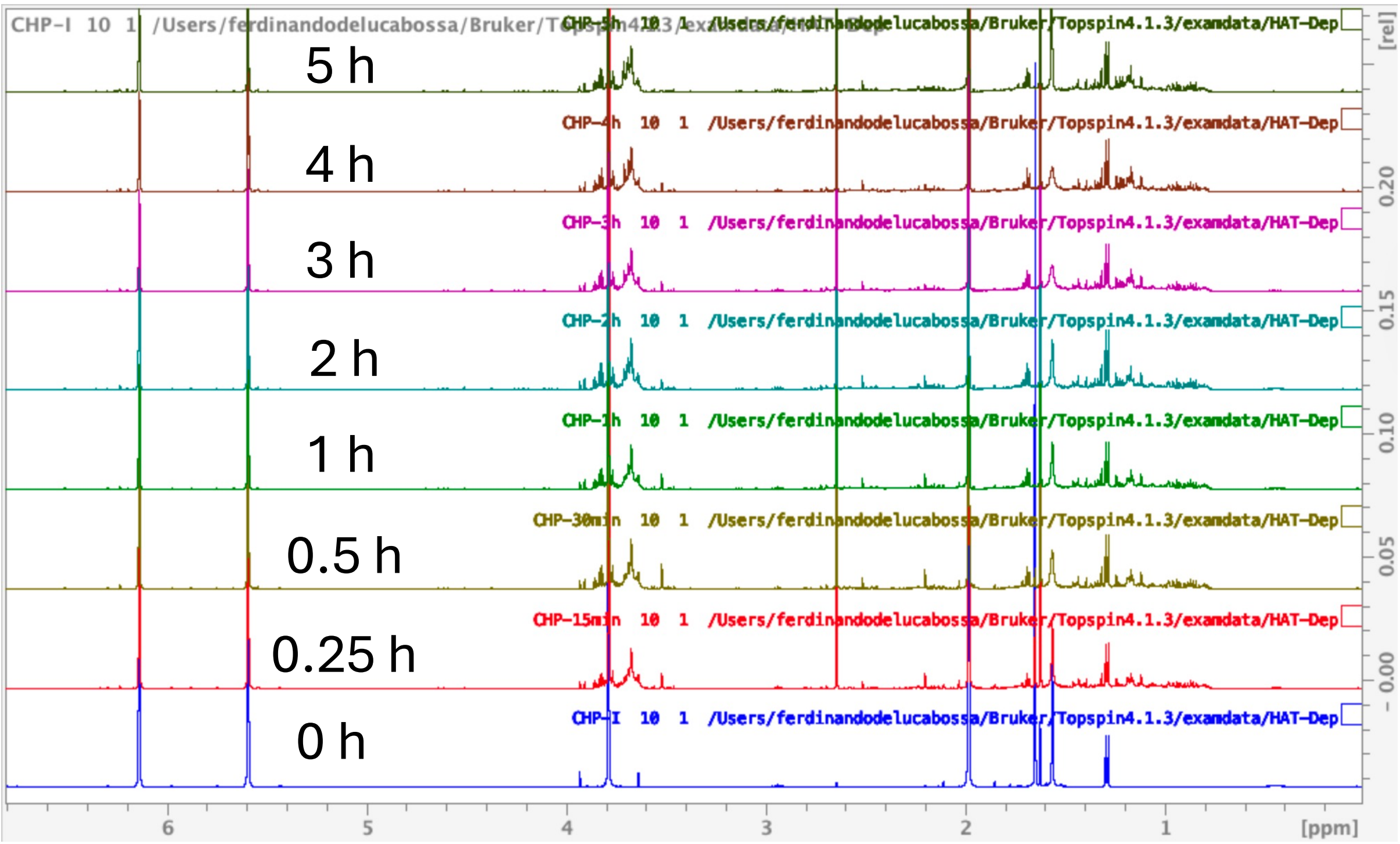

Figure S20. ^{1}H NMR spectra in $CDCl_3$ of MMA polymerization using 10 mol % [CHP]/[MMA] at 220°, and [MMA] = 912 mM (time points at 0, 0.25, 0.5, 1, 2, 3, 4, 5 h).

Gas chromatography

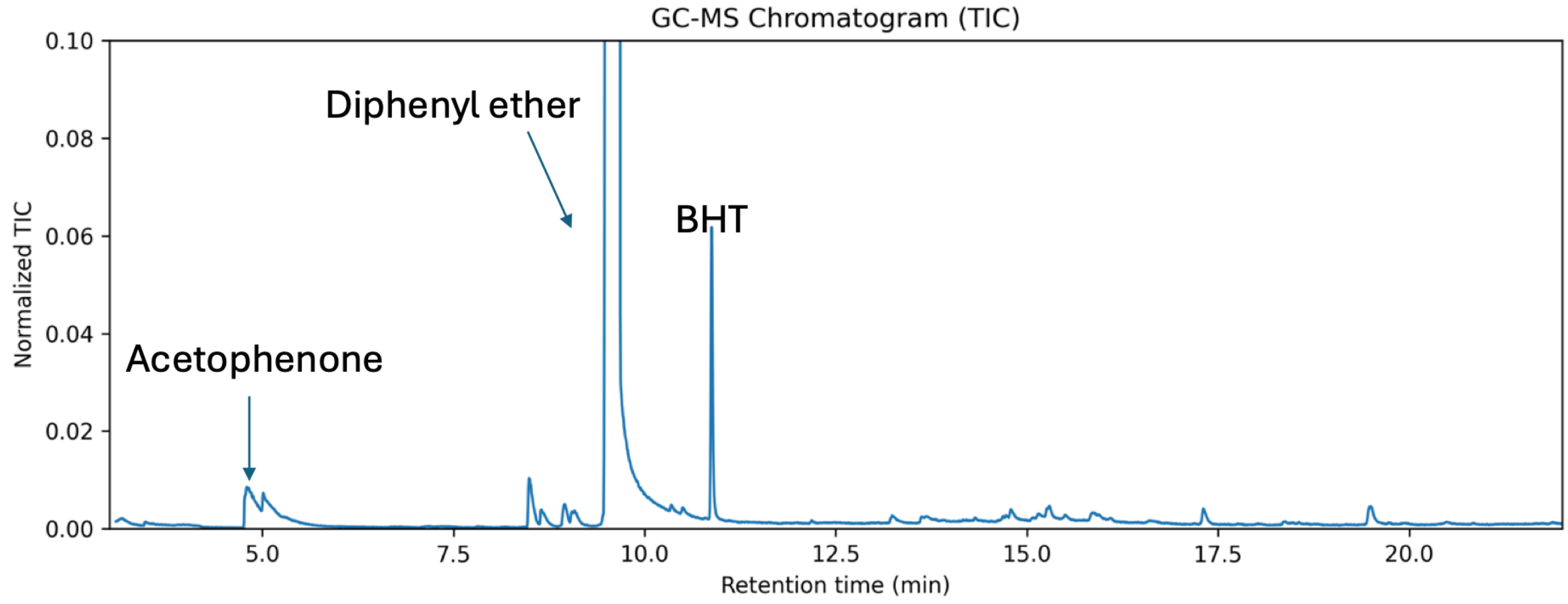

Figure S21. GC-MS Chromatogram of the reaction mixture $[CHP]/[MMA]_{RU}$ = 10 mol %, $[MMA]_{RU}$ = 912 mM after 5h at 220°C.

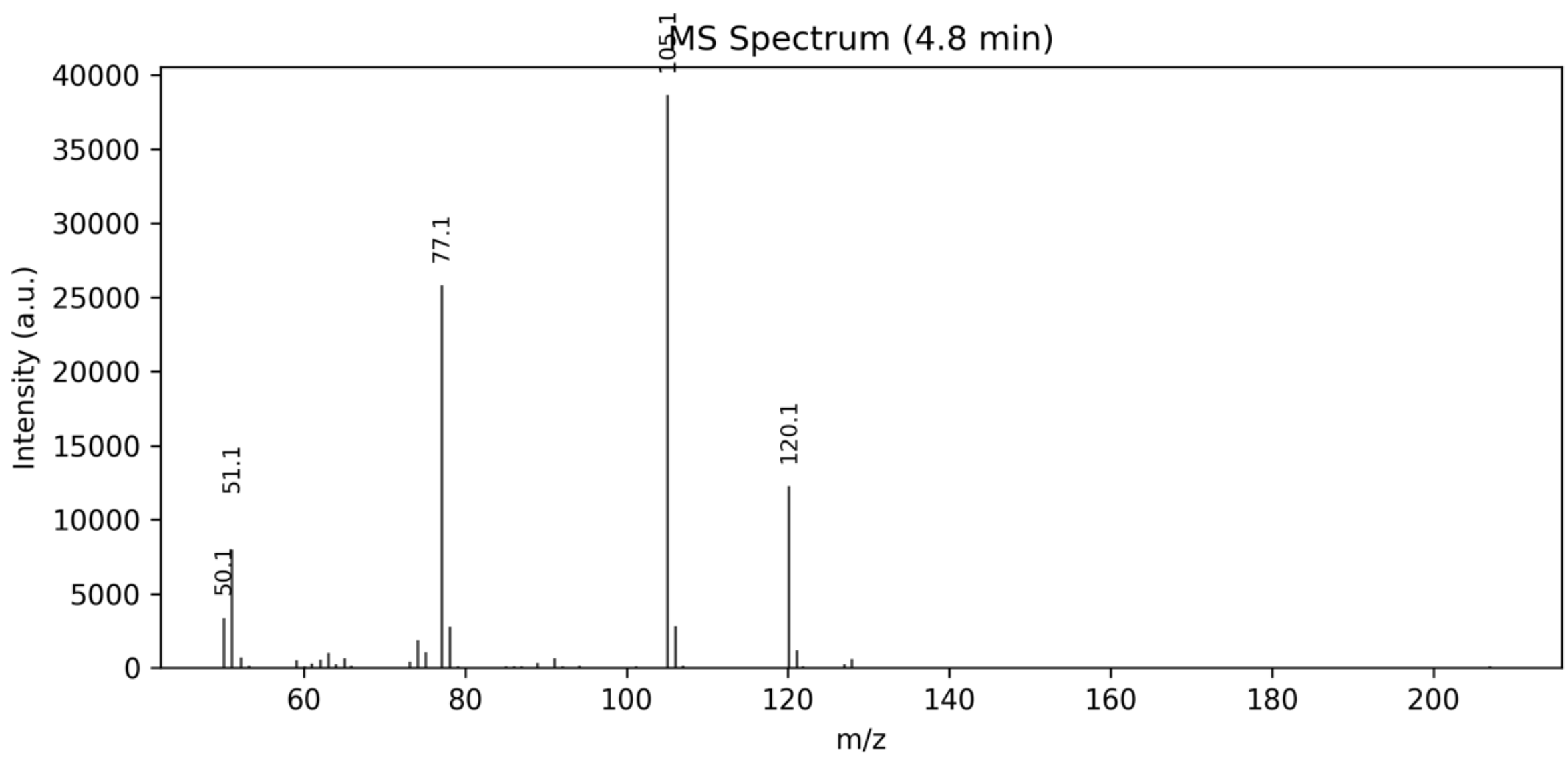

Figure S22. Mass spectrum of the peak at 5 min corresponding to acetophenone (m.w. 120.4 g/mol)

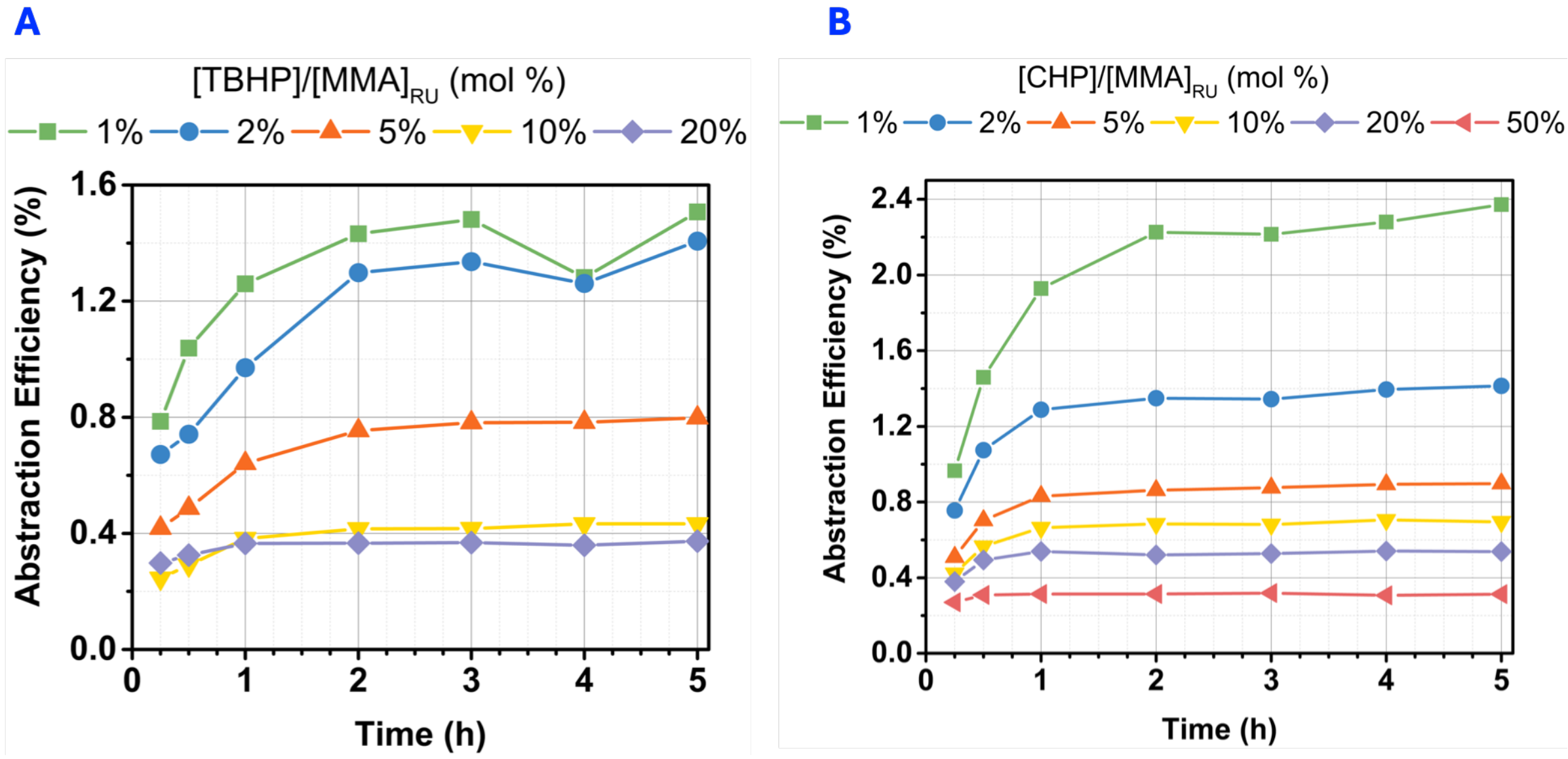

Figure S23. Abstraction efficiencies over time with different loadings of HP.

Scheme S 3. Proposed mechanism of the depolymerization of polymethacrylates using hydroperoxides as hydrogen abstractors.

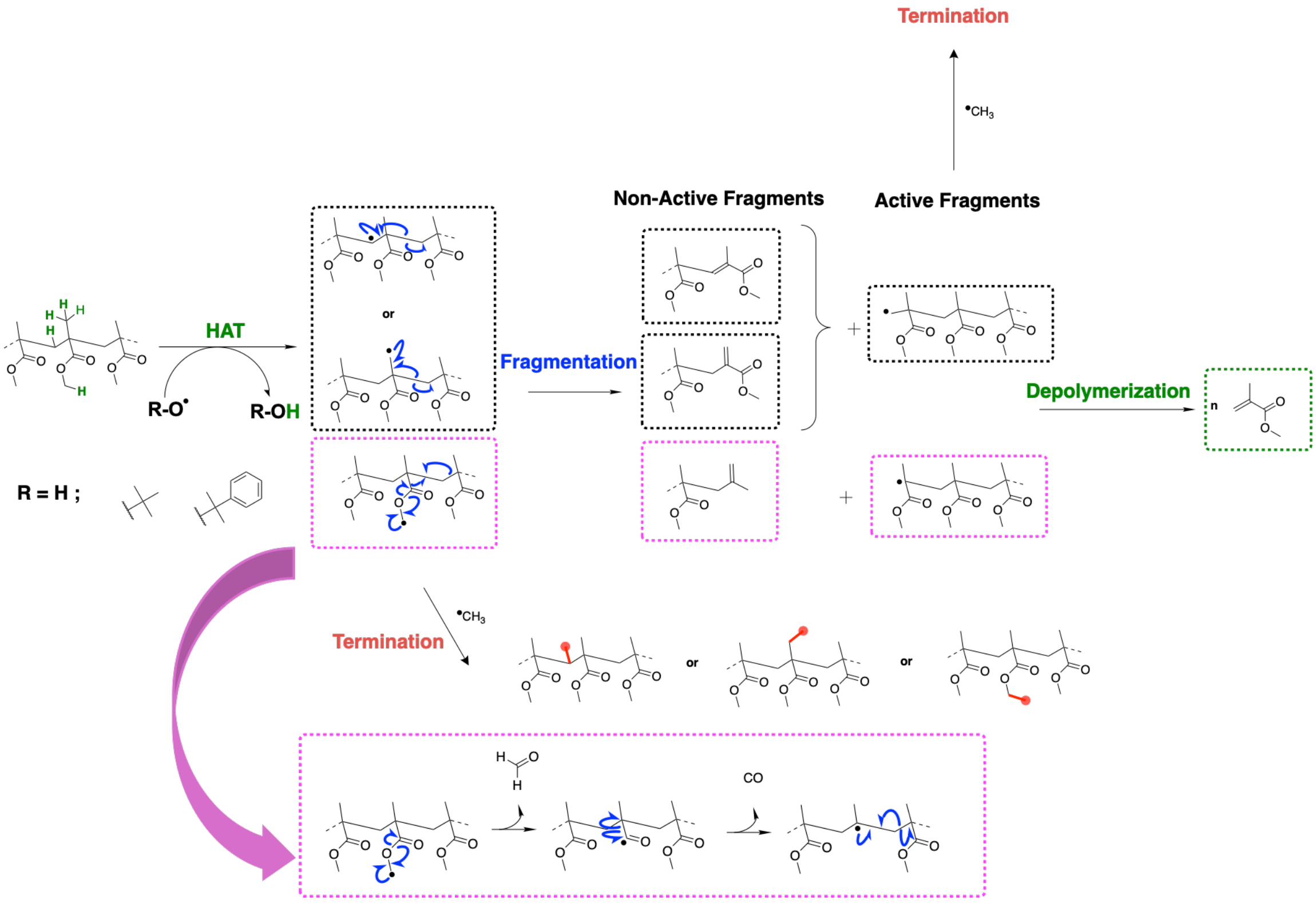

References

(1) Gruendling, T.; Junkers, T.; Guilhaus, M.; Barner-Kowollik, C. Mark–Houwink Parameters for the Universal Calibration of Acrylate, Methacrylate and Vinyl Acetate Polymers Determined by Online Size-Exclusion Chromatography—Mass Spectrometry. *Macromolecular Chemistry and Physics* **2010**, *211* (5), 520–528. https://doi.org/10.1002/macp.200900323.

(2) Roth, J. P.; Yoder, J. C.; Won, T.-J.; Mayer, J. M. Application of the Marcus Cross Relation to Hydrogen Atom Transfer Reactions. *Science* **2001**, *294* (5551), 2524–2526. https://doi.org/10.1126/science.1066130.

(3) Warren, J. J.; Mayer, J. M. Predicting Organic Hydrogen Atom Transfer Rate Constants Using the Marcus Cross Relation. *Proc. Natl. Acad. Sci. U.S.A.* **2010**, *107* (12), 5282–5287. https://doi.org/10.1073/pnas.0910347107.

(4) Mayer, J. M. Understanding Hydrogen Atom Transfer: From Bond Strengths to Marcus Theory. *Acc. Chem. Res.* **2011**, *44* (1), 36–46. https://doi.org/10.1021/ar100093z.

(5) Mayer, J. M. Simple Marcus-Theory-Type Model for Hydrogen-Atom Transfer/Proton-Coupled Electron Transfer. *J. Phys. Chem. Lett.* **2011**, *2* (12), 1481–1489. https://doi.org/10.1021/jz200021y.

(6) Katoch, A.; Mandal, D. Computational Insights into Hydrogen Atom Transfer Mediators in C–H Activation Catalysis of Nonheme Fe(IV)O Complexes. *J. Phys. Chem. B* **2025**, *129* (1), 88–95. https://doi.org/10.1021/acs.jpcb.4c05618.

(7) Blanksby, S. J.; Ellison, G. B. Bond Dissociation Energies of Organic Molecules. *Acc. Chem. Res.* **2003**, *36* (4), 255–263. https://doi.org/10.1021/ar020230d.

(8) Garwood, J. J. A.; Chen, A. D.; Nagib, D. A. Radical Polarity. *J. Am. Chem. Soc.* **2024**, *146* (41), 28034–28059. https://doi.org/10.1021/jacs.4c06774.
